# Dbp5 associates with RNA-bound Mex67 and Nab2 and its localization at the nuclear pore complex is sufficient for mRNP export and cell viability

**DOI:** 10.1371/journal.pgen.1009033

**Published:** 2020-10-01

**Authors:** Rebecca L. Adams, Susan R. Wente

**Affiliations:** Department of Cell and Developmental Biology, Vanderbilt University School of Medicine, Nashville, Tennessee, United States of America; Ohio State University, UNITED STATES

## Abstract

In *Saccharomyces cerevisiae*, the mRNA export receptor Mex67 is recruited to mature nuclear transcripts to mediate mRNA export through the nuclear pore complex (NPC) to the cytoplasm. Mex67 binds transcripts through adaptor proteins such as the poly(A) binding protein Nab2. When a transcript reaches the cytoplasmic face of the NPC, the DEAD-box protein Dbp5 acts to induce a local structural change to release Nab2 and Mex67 in an essential process termed mRNP remodeling. It is unknown how certain proteins (Nab2, Mex67) are released during Dbp5-mediated mRNP remodeling, whereas others remain associated. Here, we demonstrate that Dbp5 associates in close proximity with Mex67 and Nab2 in a cellular complex. Further, fusion of Dbp5 to Nup159 anchors Dbp5 at the cytoplasmic face of the NPC and is sufficient for cell viability. Thus, we speculate that the essential role of Dbp5 in remodeling exporting mRNPs requires its localization to the NPC and is separable from other subcellular functions of Dbp5. This work supports a model where the diverse nuclear, cytoplasmic and NPC functions of Dbp5 in the mRNA lifecycle are not interdependent and that Dbp5 is locally recruited through complex protein-protein interactions to select regions of transcripts for specific removal of transport proteins at the NPC.

## Author summary

An mRNA’s progress through the gene expression pathway is dictated by its subcellular localization and collection of proteins bound to it. At each stage in the mRNA lifecycle, members of the DEAD-box protein (Dbp) family displace specific proteins from the mRNA-protein complex (mRNP) by generating kinks in the local RNA structure, a process termed remodeling. Dbp5 is a uniquely multifunctional regulator of mRNPs, with roles in transcription, export from the nucleus, and translation. The most well understood of these functions is during mRNA export, where Dbp5 releases the transport proteins that mediate export through the nuclear pore complex (NPC). It was not known whether this function at the NPC is linked to Dbp5 actions in the nucleus, or how Dbp5 targets a specific protein for removal. Based on the results of a combination of genetic and cellular experiments, we speculate that Dbp5 remodels mRNPs at the NPC independently of its nuclear function, indicating separability of these functions. Furthermore, our data support a model wherein Dbp5 is locally recruited at the NPC through protein-protein interactions to select regions of transcripts for specific removal of transport proteins. These studies further elucidate the function of Dbp5, a regulator of mRNA fate during mRNA export and an important transition in the mRNA lifecycle.

## Introduction

The nuclear envelope (NE) barrier of a eukaryotic cell separates the two major phases of messenger (m)RNA biogenesis and translation in the gene expression pathway, ensuring that only properly processed messages reach the cytoplasm for translation. In the nucleus, mRNA is generated and transformed into an export-competent macromolecular mRNA-protein particle (mRNP) through a series of carefully surveilled mRNA processing and protein packaging steps [reviewed in 1,2]. Passage of the mature mRNP to the cytoplasm is then achieved through a highly coordinated process of transport across the nuclear pore complex (NPC), which provides a final essential point of regulation before transcripts can access the cytoplasmic translational machinery [reviewed in 3].

Following maturation in the nucleus, the complement of RNA binding proteins (RBPs) bound is dynamically and selectively exchanged throughout the mRNA life to direct each stage in the lifecycle [reviewed in 4]. For efficient transport across the NPC, mRNPs recruit the essential, conserved mRNA export receptor dimer, Mex67-Mtr2 (*S*. *cerevisiae*; Tap-p15 in humans) [5–8]. Mex67-Mtr2 interacts with unstructured phenylalanine-glycine (FG) repeats in extended domains of NPC protein components (nucleoporins, Nups) lining the central channel, thereby facilitating translocation of the mRNP across the NPC [9,10]. According to current models of mRNA export, when the mRNP reaches the cytoplasmic face, Mex67-Mtr2 is released from the transcript by the action of the RNA-dependent ATPase Dbp5 (*S*. *cerevisiae*; DDX19B in humans) [11]. At steady state, Dbp5 localization is enriched at the cytoplasmic face of the NPC through interaction with the amino (N)-terminal beta propeller of Nup159 [12–14], providing directionality to mRNA export and enabling recycling of the Mex67-Mtr2 receptor for additional export rounds. It is unknown how Mex67-Mtr2 is specifically targeted for release by Dbp5 while some other mRNP components remain associated for function in the cytoplasm.

Mex67-Mtr2 binds the mRNA through the help of adaptor proteins. The first identified adaptor, Yra1 (Aly/REF in humans), does not exhibit nucleocytoplamic shuttling in yeast *S*. *cerevisiae* [15], and is released from mRNPs at the nuclear face of the NPC after ubiquitylation by Tom1 [16]. Therefore, Yra1 is unlikely to accompany Mex67-Mtr2 with the transcript across the NPC. Conversely, the nuclear poly(A) binding protein Nab2, which forms a trimeric complex with Yra1 and Mex67 [16], shuttles across the NPC in an mRNA export-dependent manner [17–19]. Thus, Nab2 likely serves as an adaptor for Mex67 as the mRNP crosses the NPC. Indeed, Nab2 is also a target of Dbp5-mediated mRNP remodeling at the cytoplasmic face of the NPC [20].

Several other general mRNA binding proteins are loaded onto mRNA in the nucleus but are not removed during Dbp5-mediated mRNP remodeling at the NPC. For instance, the mRNA cap binding complex contains two subunits, Cbp80 (also called Sto1, Cbc1) and Cbp20 (also called Cbc2) in *S*. *cerevisiae*, that bind as the nascent transcript is capped co-transcriptionally [21]. These proteins are thought to be released during the first round of translation following mRNP export across the NPC [22]. Another RBP, the poly(A) binding protein Pab1, regulates translation and mRNA decay in the cytoplasm. The cytoplasmic localization of Pab1 is dependent on mRNA export, and models suggest it might associate with transcripts in the nucleus and remain bound after the mRNA export through the NPC [23,24]. However, using an *in vitro* mRNP remodeling assay, Dbp5 induces release of Pab1 from a short RNA substrate [20]. This indicates that an unknown mechanism exists *in vivo* to specify protein release during Dbp5-mediated mRNP remodeling.

Dbp5 belongs to the large DEAD-box family of RNA-dependent ATPases that mediate virtually every stage of mRNA metabolism [25,26]. These enzymes perform their functions through three primary modes of action: as RNA helicases to unwind short RNA-RNA or RNA-DNA duplexes, as RNP remodelers to disrupt specific protein-RNA interactions, or as stable RNA binding proteins that scaffold other interactions [reviewed in 27]. In addition to its essential function in mRNA export, Dbp5 regulates translation termination in the cytoplasm, and, notably, nuclear roles for Dbp5 and its human Dbp5 homologue DDX19B were recently described [28–33]. Structurally, DEAD-box proteins (Dbps in yeast, DDXs in humans) contain two globular RecA domains that are joined by a flexible linker [12,34,35]. Cooperative binding to RNA and ATP transitions the RecA domains from open to closed conformation, generating an extended RNA binding interface that presumably induces a kink in RNA structure. Upon interaction with RNA, ATP is hydrolyzed, and the RNA is released, resulting in non-processive activity. It is presumed that, using this mechanism, Dbp5/DDX19B locally displaces Mex67-Mtr2 from the mRNP through RNA structure changes. However, it is unknown how Dbp5 targets the specific region of RNA to modify the binding of a target protein. The interaction between RecA domains and RNA is sequence-independent, since these domains contact the phosphate backbone of RNA and not specific nucleotides [27,34]. Some DEAD-box proteins contain accessory domains to direct their activity to specific sequences; however, Dbp5 is a minimal DEAD-box protein containing only a very short, non-essential extension at its N-terminus [25]. Furthermore, Dbp5 can remodel both physiological and non-physiological RNA-protein complexes *in vitro* [20]. Therefore, it is likely that *in vivo* protein-protein interactions direct Dbp5 mRNP remodeling specificity.

The Dbp5 ATPase cycle is highly regulated at the NPC, and we have previously detailed a model of how interaction partners coordinate this cycle based on *in vivo* and *in vitro* data [36]. In this model, Dbp5 localizes to the NPC through interaction with the N-terminal beta propeller domain (NTD) of Nup159 [12,14]. Likewise, the conserved Dbp modulator Gle1 (hGle1 in humans) is enriched at the cytoplasmic face of the NPC, where it interacts with Nup42 [37–39]. Together with the small molecule inositol hexakisphosphate (IP_6_), Gle1 binds Dbp5 and stimulates ATP loading [34,40,41]. Dbp5 interaction with RNA is then speculated to induce release of Gle1, ATP hydrolysis, and mRNP remodeling [20,42]. This localized regulation would ensure that mRNP remodeling occurs at the NPC for nascently-exported transcripts and thus directs their cytoplasmic fate.

It is not yet understood what dictates the specificity of Dbp5-mediated mRNP remodeling. Based on Dbp5’s nucleocytoplasmic shuttling activity and evidence of Dbp5 bound to nuclear mRNPs, it is possible that it loads cotranscriptionally at specific sites that dictate the protein targets to be remodeled [reviewed in 43]. However, other studies assert that the specificity of mRNP remodeling is determined at the NPC via the network of remodeling complex interactions that position Dbp5 with the mRNP [12,44]. In this study, we investigated how Dbp5-mediated mRNP remodeling is directed to release specific shuttling mRNA export factors. Using a variety of approaches, we found that Dbp5 associates in an RNA-dependent manner specifically with Nab2 and Mex67, this association is observed at the NPC, and Dbp5 localization at the NPC is sufficient to direct mRNP remodeling and support cell viability. This work therefore suggests a model and mechanism wherein protein-protein interactions at the NPC dictate Dbp5 recruitment to distinct regions of mRNA for local remodeling of proteins bound to the mRNA.

## Results

### Nuclear accumulation of Dbp5 is specific to the *mex67-5* mRNA export mutant

It is unknown when during mRNA maturation that Dbp5 comes into contact with the mRNA to result in mRNP remodeling at the NPC. Dbp5 localizes to the nuclear rim at steady state. However, Dbp5 accumulates in the nucleus when the mRNA export defective *mex67-5* mutant is shifted to the restrictive temperature [45], suggesting that Dbp5 might be bound to transcripts retained in the nucleus. Based on this data, it has been suggested that Dbp5 binds the mRNP in the nucleus, exports with it, and is activated for remodeling at the cytoplasmic face of the NPC [46,47]. However, an independent role in the nucleus for Dbp5 has not been ruled out. It is possible that this process is disrupted by the *mex67-5* mutant in a way that results in higher Dbp5 nuclear localization. Alternatively, it is possible that the *mex67-5* mutant results in exposure of a nuclear Dbp5 binding interface that is not accessible under normal conditions. Therefore, we sought to address whether Dbp5 accumulation in the nucleus is a general phenotype that occurs when mRNA export is blocked or whether it is specific for the *mex67-5* mutant allele.

**Fig 1:**
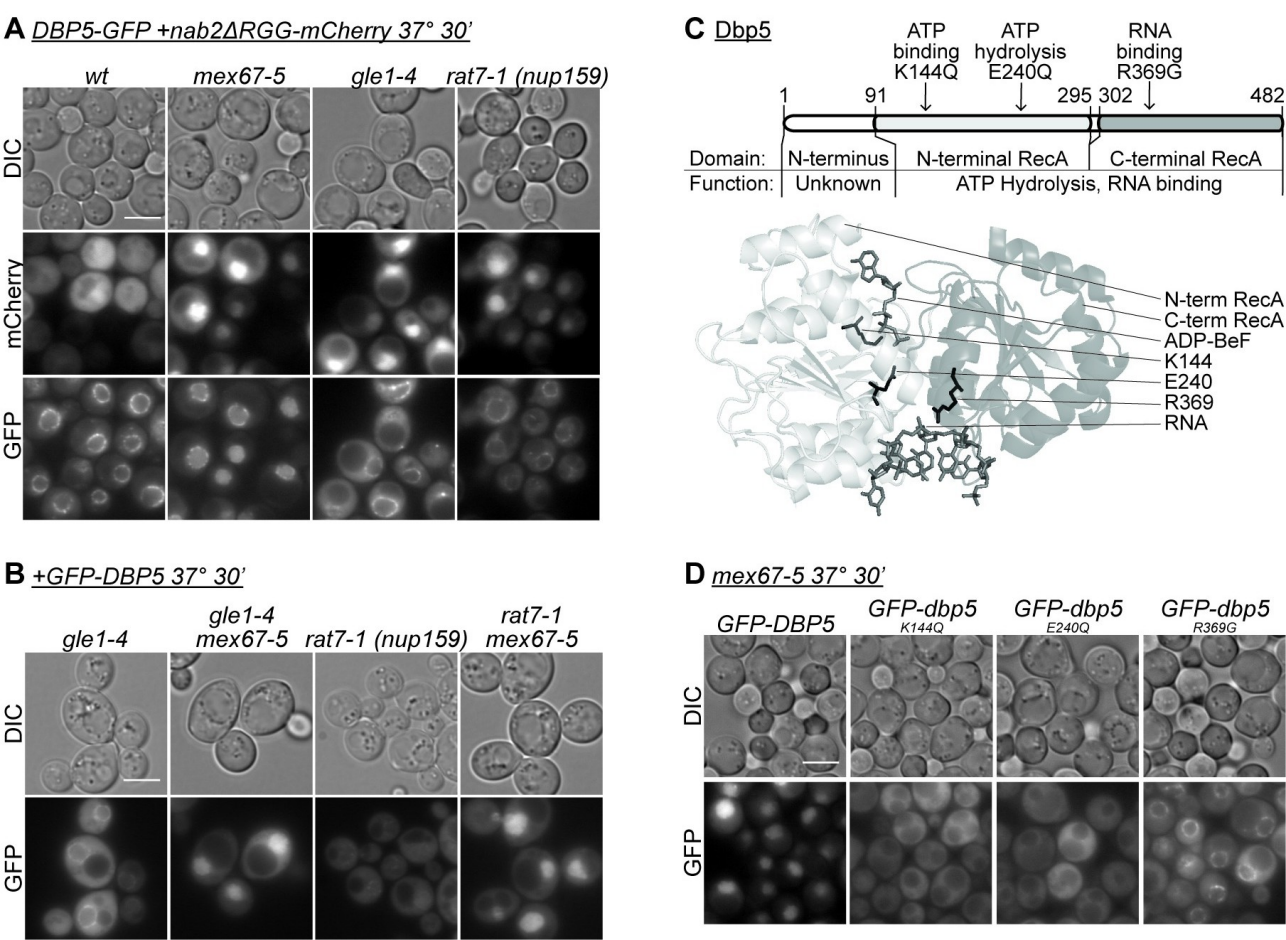
Wild-type Dbp5 accumulation in the nucleus is specific to *mex67-5*. (A) Dbp5-GFP does not accumulate in the nucleus of *rat7-1* and *gle1-4* mRNA export mutants. The indicated strains containing a *nab2ΔRGG-mCherry* plasmid were grown to mid-log phase (OD_600_~0.5) in minimal media at 23°C, shifted to 37°C for 30min, and imaged by wide-field live-cell direct fluorescence microscopy. Scale bar, 5μm. All images were adjusted identically. (B) GFP-Dbp5 accumulation in *mex67-5* is epistatic to other mRNA export mutants. The indicated strains were grown to mid-log phase (OD_600_~0.5) in minimal media at 23°C, shifted to 37°C for 30min, and imaged by wide-field live-cell direct fluorescence microscopy. Scale bar, 5μm. All images were adjusted identically. (C) Location of mutations in Dbp5 structure. Mutations mapped onto primary and tertiary structure of Dbp5 (PMID 3PEY) [34]. (D) GFP-dbp5 protein with altered ATPase cycling does not accumulate in nucleus of *mex67-5* mutants. The indicated strains were grown to mid-log phase (OD_600_~0.5) in minimal media at 23°C, shifted to 37°C for 30min, and imaged by wide-field live-cell direct fluorescence microscopy. Scale bar, 5μm. All images were adjusted identically.

Dbp5 localization was monitored in mutants that block mRNA export at different stages but under a similar experimental time frame. Whereas Mex67 is required for mRNA export from the nucleus to the cytoplasmic face of the NPC, Gle1 and Nup159 are only known to function at the terminal remodeling step, prior to cytoplasmic release of the mRNP. Temperature sensitive mutants for each of these factors, *mex67-5*, *gle1-4*, and *rat7-1 (nup159)*, demonstrate a rapid and robust poly(A)+ RNA export defect after shifting to the restrictive temperature (S1A Fig) [8,37,48]. Since *gle1-4 and rat7-1* mutants inhibit mRNA export at a step downstream of *mex67-5*, we predicted that Dbp5 would accumulate in the nucleus of all three mutants if it is bound to transcripts as they export. To assess Dbp5 localization, we integrated a GFP tag into the carboxy (C)-terminal *DBP5* coding sequence at the endogenous *DBP5* locus in each mutant strain and the wild type W303-derived strain. We also transformed a plasmid encoding nab2ΔRGG-mCherry into these strains to monitor mRNA export in the same cells. Nab2ΔRGG accumulates in the nucleus of mRNA export mutants [19,49], and is therefore a reliable reporter for a block in mRNA export. After shifting the strains to 37°C for 30 min, Dbp5-GFP strongly accumulated in the nucleus of *mex67-5*, but there was no observed accumulation in *gle1-4* or *rat7-1 (nup159)* mutants despite a block to mRNA export (Fig 1A). The same result was obtained in the absence of the *nab2ΔRGG-mCherry* reporter plasmid. To determine whether nuclear accumulation in *mex67-5* is epistatic to *gle1-4* and *rat7-1*, we generated *gle1-4 mex67-5* and *rat7-1 mex67-5* double mutant strains and analyzed exogenous GFP-Dbp5 localization. We found that mRNA export is inhibited in all strains (S1B Fig), but GFP-Dbp5 only accumulates in the nucleus of both double mutants after shifting to 37°C for 30 min (Fig 1B). This epistasis indicated that, as expected, mRNP biogenesis or export is stalled at an upstream step in *mex67-5* mutants compared to *gle1-4* and *rat7-1*. Further, the double mutant analysis demonstrated that the absence of nuclear Dbp5 accumulation in *gle1-4* and *rat7-1 (nup159)* was not due to impaired Dbp5 import or an effect of Gle1 and Nup159 on Dbp5 ATPase activity. Overall, these data were not consistent with a model where Dbp5 binds to transcripts in the nucleus to then function in mRNP remodeling at the NPC.

### Dbp5 nuclear accumulation requires ATPase and RNA binding activity

Because nuclear accumulation of Dbp5 is specific to *mex67-5*, we then analyzed whether any Dbp5 functions are required for accumulation. We transformed *mex67-5* with plasmids to express exogenous GFP-tagged wild type Dbp5 or a dbp5 variant that is functionally deficient in ATP binding (K144Q), ATP hydrolysis (E240Q), or RNA interaction (R369G) (Fig 1C) [42]. Each GFP fusion was expressed to similar levels in *mex67-5* mutants (S1 Fig). Whereas GFP-Dbp5 accumulated in the nucleus of *mex67-5* at the restrictive temperature, none of the functionally deficient GFP-dbp5 variants displayed nuclear accumulation (Fig 1D). In fact, the steady state localization of the GFP-dbp5 mutants in *mex67-5* (Fig 1D) appeared indistinguishable from their localization in a wild-type (wt) strain (S1D Fig). In parallel, the *mex67-5* mutants overexpressing GFP-dbp5 proteins were analyzed by *in situ* hybridization of oligo d(T) to assess mRNA export function, and as expected, all displayed a robust nuclear accumulation of poly(A)+ RNA under the conditions tested, indicating that exogenous Dbp5 expression did not impact the mRNA export defect (S1E Fig). It should be noted that different mechanistic reasons likely underlie each functionally deficient dbp5 variant’s failure to accumulate in a mex67-5 mutant. Nonetheless, these results demonstrate that proper ATPase cycling and RNA interaction are required for Dbp5 accumulation in *mex67-5* mutants.

### Dbp5 associates with RNA-bound Mex67 and Nab2 independent of its own RNA binding capacity

**Fig 2:**
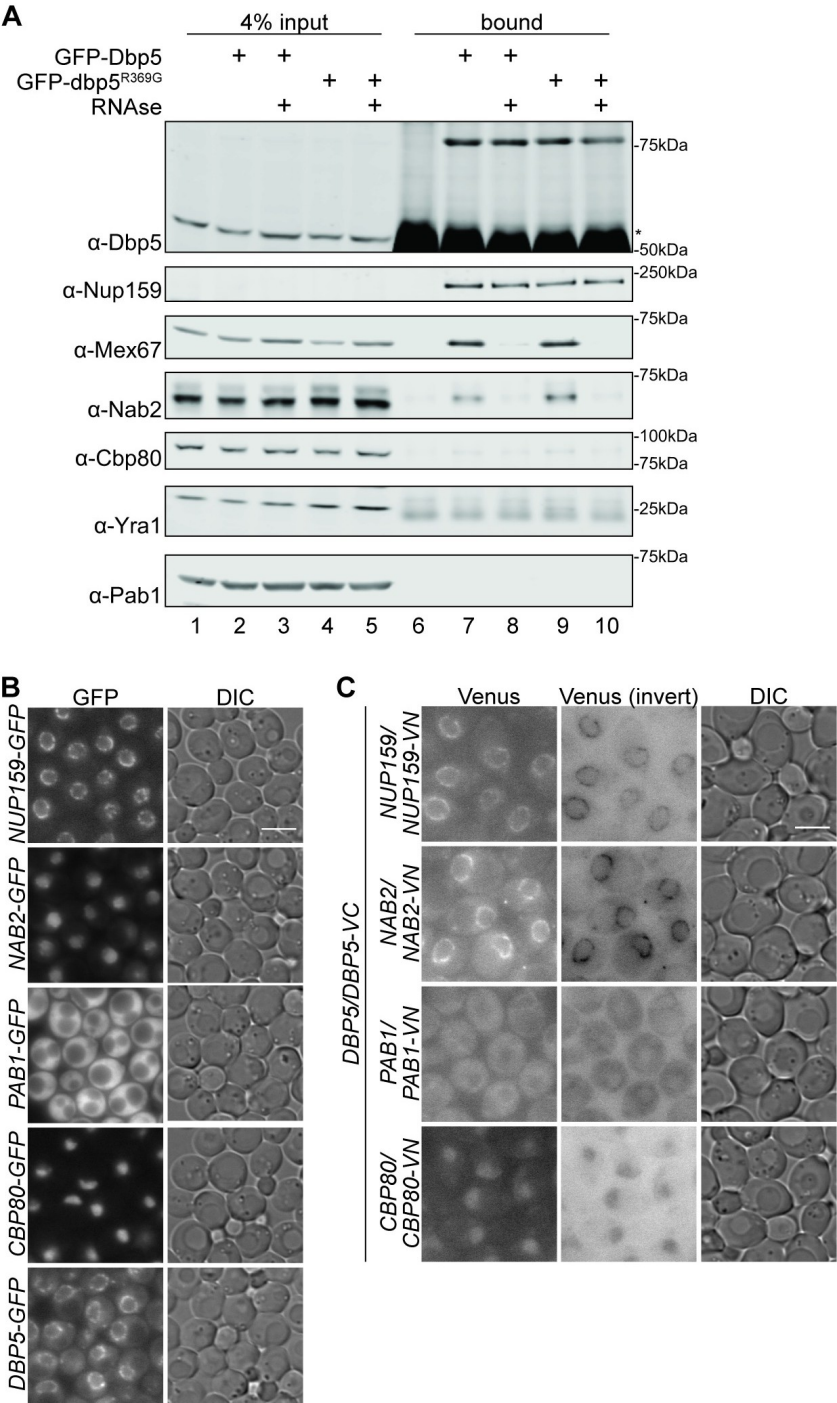
Dbp5 associates in proximity to Mex67 and Nab2. (A) Dbp5 co-precipitates Mex67 and Nab2 but no other RBPs tested. Wild type strains with *GFP-DBP5* or *GFP-dbp5*^*R369G*^ vectors were grown to mid-log phase in minimal media. Cells were lysed, treated with RNase A or water, and GFP-tagged proteins were immunoprecipitated with anti-GFP antibodies. Eluates were resolved by SDS-PAGE and immunoblotted with the indicated antibodies. Asterisk indicates antibody heavy chain. (B) Localization of GFP-tagged RBPs and Nup159. Indicated haploid strains were grown to mid-log phase (OD_600_~0.5) at 23°C and imaged by wide-field live-cell direct fluorescence microscopy. Scale bar, 5μm. (C) Dbp5 BiFC association with Nab2 is observed at the NPC. Indicated heterozygous diploid strains were grown to mid-log phase (OD_600_~0.5) at 23°C and imaged by wide-field live-cell direct fluorescence microscopy. Scale bar, 5μm. All DIC and Venus images were adjusted identically.

For Dbp5 to specifically remodel Mex67-Mtr2 and Nab2 from mRNPs, we speculated that Dbp5 would be recruited proximal to these factors on the mRNA. Indeed, Dbp5 associates with Mex67 in an RNase-sensitive manner based on co-immunoprecipitation studies [11]. However, it is unclear whether the RNase-sensitivity of the reported Dbp5-Mex67 association reflects an RNA binding requirement for Dbp5, Mex67, or both. To test whether Dbp5 interaction with RNA is required for association with Mex67, we utilized the GFP-dbp5^R369G^ variant, which has <5% affinity for RNA compared to wild type Dbp5 [42]. Exogenously expressed GFP-tagged Dbp5 or dbp5^R369G^ was immunoprecipitated from wild type cells, and co-purifying proteins were analyzed by Western blot. Mex67 displayed equivalent co-isolation with GFP-Dbp5 and GFP-dbp5^R369G^, demonstrating that Dbp5 interaction with RNA is not required for its association with Mex67 (Fig 2A). This co-isolation might represent a direct interaction or association within a complex. As previously determined, the Dbp5-Mex67 association was sensitive to treatment with RNase [11], whereas Dbp5 interaction with Nup159 was not. We then assessed whether Dbp5 associates with other RBPs in similar fashion. Both GFP-Dbp5 and GFP-dbp5^R369G^ co-precipitated Nab2 in an RNase-sensitive manner; however, Yra1, Pab1, or Cbp80 were not detected (Fig 2A). This revealed that the co-isolations of Dbp5 with RNA-bound Mex67 or Nab2 were specific under these conditions. Furthermore, the lack of co-immunoprecipitation of other tested RBPs suggests that closely associated protein complexes were captured by this approach and not whole mRNPs. Since RNA is required for the association of Dbp5 with Mex67 and Nab2, but Dbp5 interaction with RNA is not, these results suggest that Mex67 and/or Nab2 interaction with RNA permits binding of Dbp5 to the mRNP. We anticipate that interaction with RNA in the context of an mRNP imparts either a conformational change in Nab2 or Mex67 or an additional unknown protein-protein interaction that permits recruitment of Dbp5, whether directly or indirectly. Allowing for the potential that post-lysis associations can be captured or lost during this experiment, these results provided promising support of specific Dbp5-Mex67 or Dbp5-Nab2 association but prompted further *in vivo* examination.

### Dbp5-Nab2 BiFC signal is observed at the NPC

To further understand how Dbp5, Mex67, and Nab2 association contribute to mRNA export, we employed bimolecular fluorescence complementation (BiFC) to examine the steady state co-localization of such complexes. Using the split Venus approach, two putative binding partners (or closely associating proteins in a complex) are fused to either the N-terminal (VN) or C-terminal domains (VC) of the Venus fluorophore. If the tagged proteins associate in close proximity, the Venus fluorophore is able to properly fold, and after a delay needed for chromophore maturation, fluorescence can be detected [reviewed in 50]. A positive fluorescent signal indicates proximal association between the two proteins within the range of a few nanometers [reviewed in 51], indicating either a direct interaction or close association within a complex. Additionally, the location of the florescent signal indicates the steady state subcellular localization of the VN-VC complex, although chromophore maturation time and VN-VC dimer stability impacts interpretation of this localization with respect to where the native protein interactions occur. We anticipated that the association of RBPs with Dbp5 is very transient, and this approach would provide a means to capture such short-lived complexes *in vivo*, with the caveat that protein associations may not be detected in their endogenous time frame but can be locked together over a longer time period [reviewed in 50,51].

We first generated haploid strains in which the protein of interest is tagged at the C-terminus with either the Venus N-terminal (VN) or C-terminal (VC) domains. Tagging Mex67, Mtr2, and Yra1 resulted in loss of viability, and these fusions were not pursued further in heterozygous diploid strains due to the potential for functional perturbations of the fusion protein. Viable tagged Dbp5, Nup159, Nab2, Pab1, and Cbp80 haploid strains did not display fluorescent signal (S2A Fig) and were mated to generate heterozygous diploid, doubly tagged strains for experimental analysis. Although the results shown represent VC-tagged Dbp5 and VN-tagged candidate binding proteins, reciprocal tagging confirmed the observed association. To compare the localization of the specific split Venus interactions to wild-type protein localization, the fluorescent signal of GFP-tagged haploid strains was analyzed in parallel (Fig 2B). Western blotting confirmed the correct genotype of all tested strains (S2B Fig). Split Venus diploid and GFP-tagged haploid strains were grown to mid-log phase at 23°C, and steady state localization was determined by wide-field live-cell direct fluorescence microscopy (Fig 2B and 2C). Interaction between Dbp5-VC and Nup159-VN served as a positive control, with split Venus signal apparent at the nuclear rim (Fig 2C). Importantly, the *DBP5-VC NAB2-VN* strain exhibited a robust split Venus signal at the nuclear rim (Fig 2C), whereas Nab2-GFP was nuclear localized (Fig 2B), as previously documented [18,52]. This result further substantiated that Dbp5-Nab2 associate in close proximity, and pinpointed the steady state localization of this association at the NE. In contrast, only weak nucleoplasmic fluorescence was produced in the Dbp5-VC/Cbp80-VN split Venus strain and no signal above background fluorescence was detected between Dbp5-VC and Pab1-VN.

### NPC-localized Dbp5-VC and Nab2-VN association is lost during the stress response and when cytoplasmic FG domains are deleted

**Fig 3:**
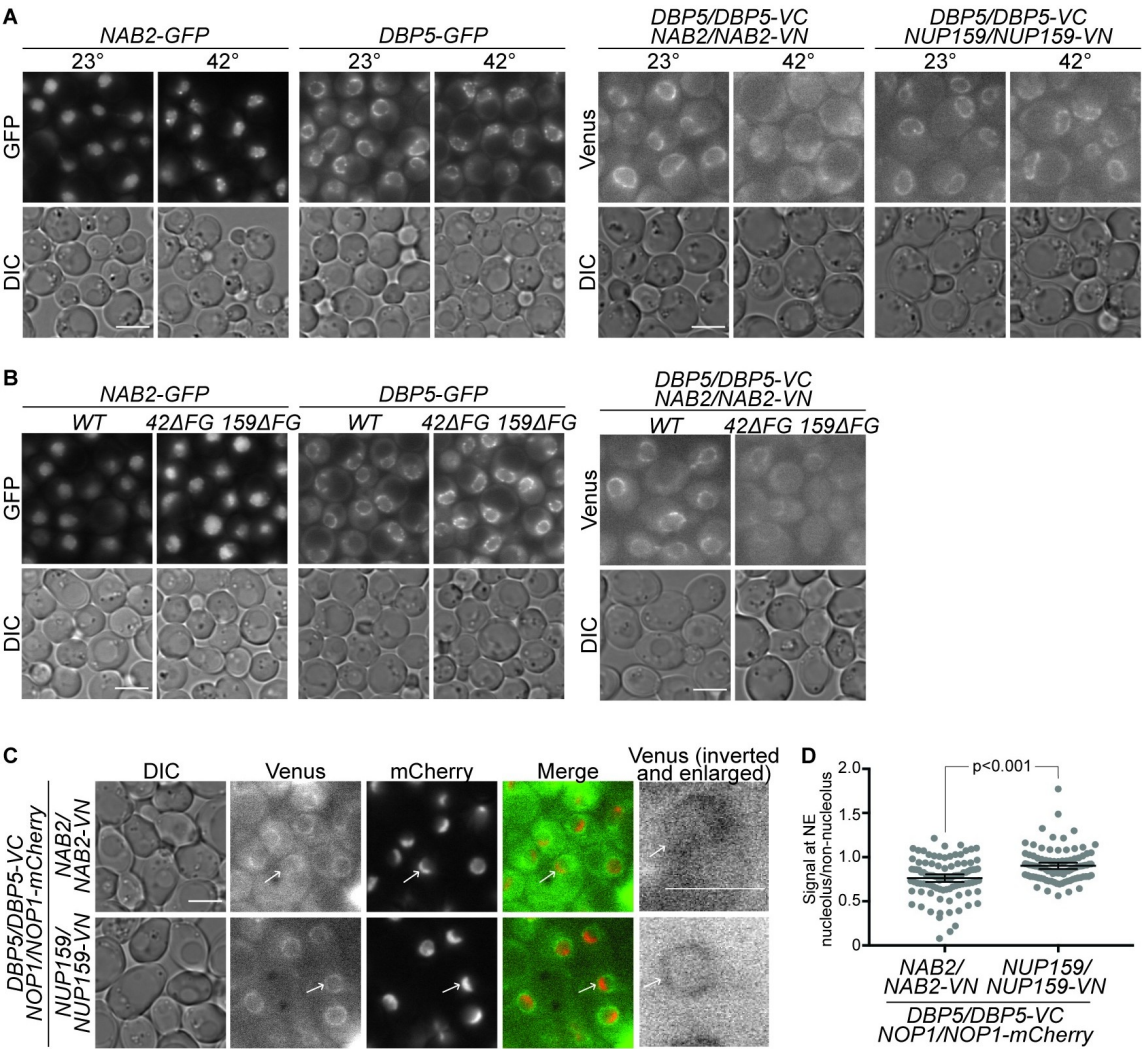
Dbp5-VC and Nab2-VN split Venus signal occurs during mRNA export under normal conditions. (A) The Dbp5-VC and Nab2-VN split Venus signal is disrupted during heat shock. Indicated strains were grown to mid-log phase (OD_600_~0.5) at 23°C, shifted to 42°C for 10min, and imaged by wide-field live-cell direct fluorescence microscopy. Scale bar, 5μm. All images were adjusted identically. (B) Deletion of Nup159 and Nup42 FG domains reduces Dbp5 and Nab2 association *in vivo*. Indicated strains were grown to mid-log phase (OD_600_~0.5) at 23°C and imaged by wide-field live-cell direct fluorescence microscopy. Scale bar, 5μm. All images were adjusted identically. (C & D) Under conditions of robust ribosome export, the Dbp5 and Nab2 association occurs at NPCs opposite the nucleolus. (C) Indicated strains were grown to saturation overnight at 30°C, diluted to early-log phase (OD_600_~0.2) at 23°C, grown for 7 hrs, and imaged by wide-field live-cell direct fluorescence microscopy. Arrows indicate nucleolar region in select cells. Panels on the right display the single cell with the arrow in previous panels, enlarged to show detail of split Venus signal. Scale bar, 5μm. (D) Quantification of Venus signal as described in Methods and depicted in S4 Fig. P-value result from t-test.

During heat stress, bulk mRNA export is inhibited and Mex67 is co-transcriptionally loaded onto heat shock mRNA transcripts independent of known adaptor proteins [53–56]. As part of this response, Nab2 is phosphorylated and redistributes to intranuclear foci [57]. To determine if heat shock impacts the Dbp5-Nab2 association at the NE, we analyzed the split Venus interactions with Dbp5-VC following a shift to 42°C for 10 minutes. We found that the split Venus signal between Dbp5-VC and Nab2-VN at the NE was perturbed under these heat shock conditions, and no increased signal was observed in the nucleus (Fig 3A, S3A Fig). In contrast, fluorescence resulting from interactions between Dbp5-VC and Nup159-VN was unchanged. This demonstrated that Nab2 does not localize in proximity to Dbp5 at the NPC during the initial stages of heat shock, consistent with it being absent from exporting heat shock mRNPs; therefore, Nab2 is not a remodeling substrate of Dbp5 under these conditions. Furthermore, Dbp5 did not redistribute with Nab2 during stress to nuclear foci. Taken together, these data highlighted the specificity of the Dbp5-VC Nab2-VN interaction at the NE.

Based on our previous work demonstrating a role for the FG domains of Nup42 and Nup159 during mRNP remodeling [44], we next analyzed whether Dbp5-VC is capable of localizing in proximity to Nab2-VN in a *nup42ΔFG nup159ΔFG* homozygous diploid strain. Fluorescence indicating a split Venus interaction between Dbp5-VC and Nab2-VN was substantially reduced in the *nup42ΔFG nup159ΔFG* background despite the observation that Dbp5-GFP remains localized to the NE in this background (Fig 3B, S3B Fig). This supported our previous model wherein we proposed that the Nup42 and Nup159 FG domains bind Mex67-Mtr2 to position the mRNP for interaction with Dbp5 and remodeling.

A major caveat in interpreting BiFC data is that complementation of fluorophore folding can lock two interacting proteins together over timescales longer than their natural interaction. Furthermore, once the fluorescent protein is completely folded, it can take on the order of minutes to hours for the fluorophore to mature and produce a fluorescent signal [reviewed in 51]. Therefore, although this approach can demonstrate where the BiFC signal occurs in a cell, the association shown does not reflect normal interaction kinetics, and there exists a delay between the initial association and observed fluorescence. Likely due to an effect on interaction kinetics, we observed that, although haploid individually tagged *DBP5-VC* and *NAB2-VN* strains are viable, a haploid *DBP5-VC NAB2-VN* is inviable. Moreover, the Dbp5-VC and Nab2-VN association in the heterozygous diploid isn’t reversible under conditions where active mRNA export is blocked (S3C Fig). Specifically, after a 30-minute treatment with thiolutin, which blocks RNA polymerase activity [58] and halts RNA export activity [59], the Dbp5-VC Nab2-VN BiFC signal was not reduced in many cells, and some cells displayed increased signal (S3C Fig). Confirming that mRNA export was blocked by thiolutin treatment, the nab2ΔRGG-mCherry mRNA export reporter accumulated in the nucleus in the time points analyzed. Notably, a slight increase in the Dbp5-VC Nup159-VN BiFC signal was also observed (S3C Fig). However, despite the potential for altered interaction kinetics, this assay demonstrates a proximal association between Dbp5 and Nab2 *in vivo*.

### Dbp5-VC and Nab2-VN association is observed at NPCs opposing the nucleolus

Under the conditions tested, the Dbp5-VC Nab2-VN split Venus signal was observed roughly around the entire nuclear rim; however, some cells appeared to display disproportionate signal around portions of the NE. Other prior studies propose that mRNP and ribosome export occurs only through distinct NPCs, based on the observation that the mRNA quality control proteins Mlp1, Mlp2, and Pml39 localize specifically to NPCs opposite the nucleolus [60,61]. In contrast, other recent advanced imaging studies suggest that exporting mRNPs distribute to NPCs equally around the NE [62,63]. In order to further differentiate between these models, we analyzed the split Venus signal between Dbp5-VC and Nab2-VN under conditions that induce robust ribosome biosynthesis and export [64,65]. Briefly, cells grown to stationary phase at 30°C were diluted to early log phase and cultured at 23°C for 7 hours (approximately 2.5 doubling times) prior to imaging. To denote the location of the nucleolus in each strain, sequence encoding an mCherry tag was integrated into the coding sequence of the nucleolar Nop1 protein to generate a C-terminal fusion [66]. Following induction of ribosome biogenesis, Dbp5-VC Nab2-VN split Venus signal was reduced at locations around the NE that were enriched for Nop1-mCherry signal (Fig 3C). It should be noted that an overlap of the mCherry and split Venus signal was observed in a small number of cells, likely attributable to an artifact of widefield imaging. The opposing patterns of Venus and Nop1-mCherry fluorescence were not observed in the *DBP5-VC NUP159-VN* strain. Indeed, quantification of the Venus signal demonstrated reduced fluorescence adjacent to Nop1-mCherry relative to the remainder of the NE in *DBP5-VC NAB2-VN* strains but not in *DBP5-VC NUP159-VN* strains (Fig 3D, S3E Fig). This supported the hypothesis that a distinct subset of NPCs might be specified for export of mRNA and ribosomes, potentially based on the association of quality control machinery with the NPC nuclear basket.

### A Dbp5-Nup159 fusion is stably associated with the NE

The data presented thus far suggests the possibility that Dbp5 comes into contact with Nab2- and Mex67-bound mRNPs at the NPC, and not prior to export. To formally test whether nuclear Dbp5 is essential for mRNP remodeling, we sought to determine if nuclear localization of Dbp5 is required. We predicted that if Dbp5 must interact with mRNA in the nucleus to enable subsequent mRNP remodeling at the NPC, the loss of Dbp5 nuclear localization would abolish mRNP remodeling and result in cell death. Initial attempts to locate and alter a putative nuclear localization signal (NLS) for Dbp5 were unsuccessful; therefore, we employed a tethering approach to anchor Dbp5 exclusively at the cytoplasmic face of NPC.

**Fig 4:**
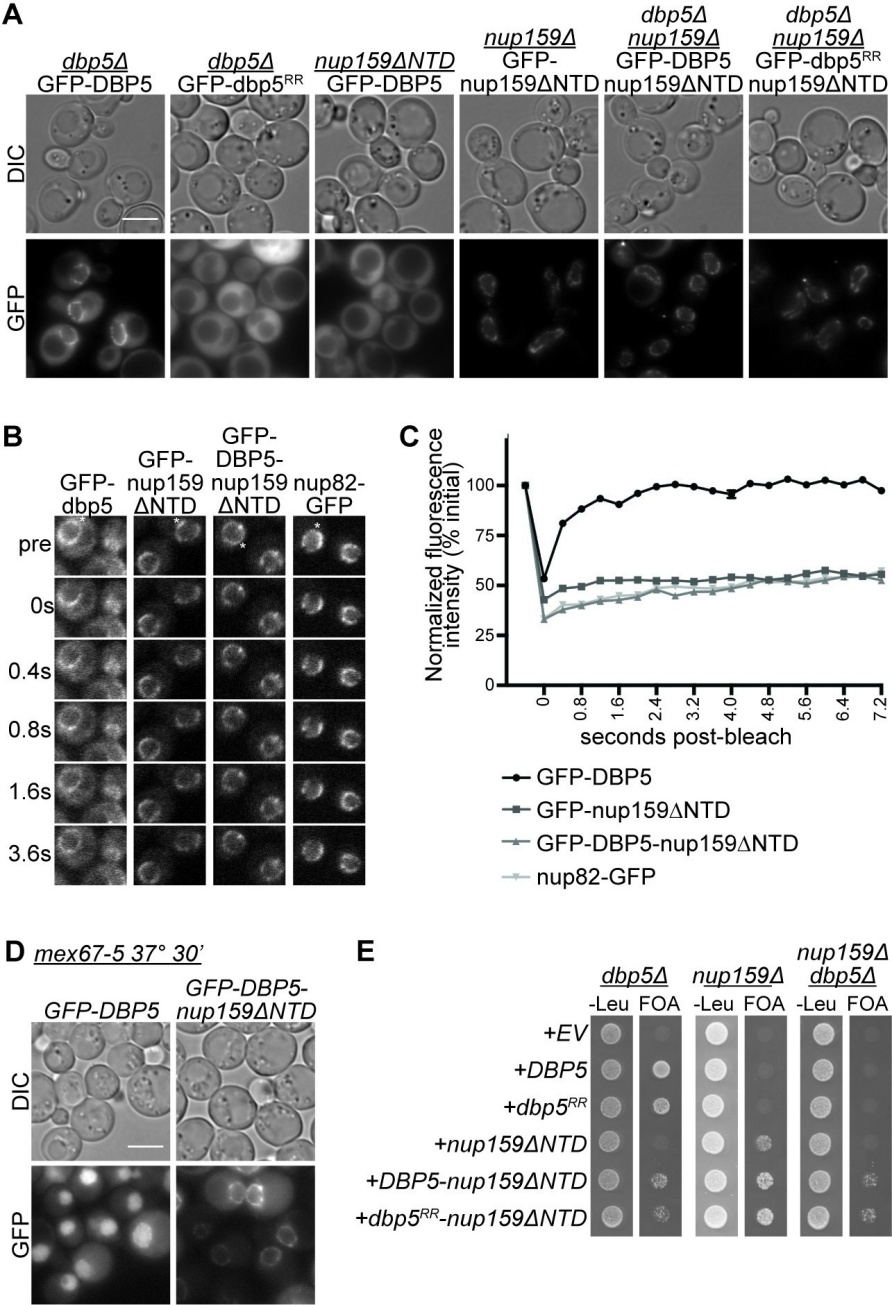
Dbp5 localization at the NPC is sufficient for viability. (A) The Dbp5-nup159ΔNTD fusion protein is localized at the NPC. Indicated strains were grown to mid-log phase (OD_600_~0.5) at 23°C and imaged by wide-field live-cell direct fluorescence microscopy. Scale bar, 5μm. Images of GFP-Dbp5, GFP-nup159ΔN, GFP-Dbp5-nup159ΔN, and GFP-dbp5^RR^-nup159ΔN were adjusted identically. (B & C) GFP-Dbp5-nup159ΔNTD is stably localized to the NPC. WT strains containing the indicated GFP vectors were grown to mid-log phase (OD_600_~0.5) at 23°C. FRAP analysis was performed by photobleaching a small rectangle-shaped area of the nuclear envelope (indicated by *) with measurement of fluorescent recovery every 400ms for 7.2s. Representative images are shown in (B) and quantification of relative fluorescence is shown in (C). (D) GFP-Dbp5-nup159ΔNTD does not accumulate in the nucleus of *mex67-5* mutants. *mex67-5* strains transformed with the indicated GFP vectors were grown to mid-log phase (OD_600_~0.5) at 23°C, shifted to 37°C for 30min, and imaged by wide-field live-cell direct fluorescence microscopy. Scale bar, 5μm. Images were adjusted identically. (E) *DBP5-nup159ΔNTD* rescues inviability of *dbp5Δ nup159Δ* mutants. Strains bearing the indicated gene deletions in addition to *DBP5/URA* and/or *NUP159/URA* vectors were spotted onto–Leu or 5-FOA at 23°C. Growth on 5-FOA indicates rescue of lethality.

Although GFP-Dbp5 is enriched at the cytoplasmic face of the NPC at steady state (Fig 4A) [12], this interaction is highly dynamic, with a reported half-life at the NE of ~0.8s in *S*. *cerevisiae* [42] and 55ms at the NPC in *Chironomus tentans* [67]. Conversely, Nup159 is stably associated with the NPC, based on a residence time of 44 hours measured for its mammalian homologue, Nup214 [68]. Deletion of the Nup159 NTD results in a loss of GFP-Dbp5 enrichment at the nuclear rim (Fig 4A) [45]. To stably anchor Dbp5 to the cytoplasmic face of the NPC, we therefore generated a fusion between Dbp5 and Nup159 (*DBP5-nup159ΔNTD)*, where the Dbp5 binding site on Nup159 is deleted. We reasoned that deletion of the endogenous Dbp5 binding site on Nup159 would be essential for interpreting subsequent localization and dynamics. In addition to localizing Dbp5 to the NE, the Nup159NTD is also required for efficient Dbp5 remodeling [40], and a *dbp5*^*RR*^ allele that exchanges ADP more readily than wild type Dbp5 bypasses this requirement. If the *DBP5-nup159ΔNTD* fusion further impairs the Dbp5 ATPase cycle (compared to *nup159ΔNTD* alone), this would confound our interpretation of the requirement for nuclear Dbp5 localization. Thus, we also generated a *dbp5*^*RR*^*-nup159ΔNTD* fusion allele as a control for Nup159-associated effects on mRNA export.

We first generated GFP-tagged alleles of the Dbp5-nup159ΔNTD fusions to confirm localization to the nuclear rim. GFP-Dbp5-nup159ΔNTD was detectable as a full-length protein from cell lysate (S4A Fig). A smaller GFP fusion was detected in the *GFP-DBP5-nup159ΔNTD* mutant cell lysate. We reasoned that this is a degradation product from post-lysis proteolysis of the Nup159 FG domain, which is commonly observed with FG nups. Live cell imaging of GFP-Dbp5-nup159ΔNTD and GFP-dbp5^RR^-nup159ΔNTD confirmed steady state localization for the fusion protein at the NE (Fig 4A) with no detectable nuclear accumulation. The steady state localization of the GFP-Dbp5-nup159ΔNTD fusion suggested that the majority of GFP fusion protein is full-length in cells. In a few cells, we observed a low pan-cellular GFP signal in *GFP-nup159ΔNTD*, *GFP-Dbp5-nup159ΔNTD*, and *GFP-dbp5*^*RR*^*-nup159ΔNTD* strains. We speculated that this signal represents cell-to-cell variability in protein levels due to plasmid-based expression. We also analyzed the dynamics of nuclear rim localization by determining the fluorescence recovery after photobleaching (FRAP) for GFP-Dbp5, GFP-nup159ΔNTD and GFP-Dbp5-nup159ΔNTD. Nup82-GFP served as a control for stable association at the NPC, as previously shown [42]. As previously determined, signal from GFP-Dbp5 that is not fused to nup159ΔNTD recovers rapidly after photobleaching, indicating dynamic association with the NPC (Fig 4B and 4C) [42,69]. However, neither GFP-nup159ΔNTD nor GFP-Dbp5-nup159ΔNTD fluorescence recovered from photobleaching over the time course analyzed, instead displaying dynamics similar to Nup82-GFP. The stable NE association of the GFP signal in the GFP-Dbp5-nup159ΔNTD cells further indicated that in vivo degradation (and release of a free GFP-Dbp5) is unlikely.

To determine whether the Dbp5-nup159ΔNTD fusion remains stably associated with the NPC even in response to defective Mex67 function and the resulting mRNA export defect, we analyzed localization of exogenously expressed GFP fusions in *mex67-5* mutants that also express wild-type copies of *DBP5* and *NUP159* from their endogenous loci. Although GFP-Dbp5 accumulated in the nucleus of *mex67-5* mutants at the nonpermissive temperature, GFP-Dbp5-nup159ΔNTD exhibited nuclear rim localization with no observed redistribution to the nucleus (Fig 4D). A lack of nuclear GFP accumulation is also observed in cells that have higher levels of GFP-Dbp5-nup159ΔNTD expression, indicating that there is not a functional pool of GFP-Dbp5 generated from this fusion. This demonstrates that the Dbp5-nup159ΔNTD fusion protein is stably associated with the NPC, and there is no observable pool of Dbp5 that accumulates in the nucleus from this fusion.

### Anchoring Dbp5 at the NPC is sufficient for viability

We reasoned that if Dbp5 association with mRNA is required prior to export, a strain expressing the Dbp5-nup159ΔNTD fusion as the only source of Dbp5 would be inviable. To determine if the fusion proteins supported viability, we transformed empty vector (EV), *DBP5*, *dbp5*^*RR*^, *nup159ΔNTD*, *DBP5-nup159ΔNTD*, and *dbp5*^*RR*^*-nup159ΔNTD* plasmids into *nup159Δ*, *dbp5Δ*, or *nup159Δ dbp5Δ* shuffle strains. Strikingly, *DBP5-nup159ΔNTD* and *dbp5*^*RR*^*-nup159ΔNTD* plasmids rescued lethality of *nup159Δ*, *dbp5Δ*, and *nup159Δ dbp5Δ* (Fig 4E). Furthermore, expression of the fusions of either Dbp5 or dbp5^RR^ to nup159ΔNTD supported more robust growth than nup159ΔNTD alone (S4B Fig), and GFP-tagged strains exhibited growth similar to untagged strains (S4C Fig),

Although the *DBP5-nup159ΔNTD* strain is viable, it does have a growth defect compared to strains expressing wild-type Dbp5 (S4B Fig). In order to assess whether this growth defect is due at least in part to an mRNA export defect, we performed *in situ* hybridization to analyze poly(A)+ RNA localization. Reflecting the growth defect in this strain, *DBP5-nup159ΔNTD* results in an accumulation of mRNA in the nucleus compared to wild-type cells at 23°C, (S4D and S4E Fig). However, the level of steady-state mRNA in the cytoplasm of *DBP5-nup159ΔNTD* mutants is comparable to that of wild-type cells, indicating that Dbp5 anchored to the NPC is sufficient for mRNA export. An increased mRNA export defect was observed *nup159ΔNTD* at higher temperatures, as previously reported [14]. Together, these studies indicated that Dbp5 localization to the cytoplasmic face of the NPC is the sole site of Dbp5’s essential function in mRNA export.

## Discussion

Given the recently described nuclear role for the human Dbp5 homologue DDX19B [32] and the suggested function for Dbp5 during tRNA processing in the nucleus [33], a more detailed knowledge of Dbp5’s mode and site(s) of interaction with the mRNP is critical to understanding Dbp5 functions. In this study, we present several lines of evidence that Dbp5 association with the mRNP prior to export is not required for its remodeling activity at the NPC. First, a *DBP5*-*nup159ΔNTD* fusion that restricts Dbp5 from the nucleus is viable and stably associated with the NPC (Fig 4), demonstrating that nuclear Dbp5 is not required for the essential process of mRNP remodeling. Second, robust bimolecular fluorescence between split Venus-tagged Dbp5 and Nab2 was detected at the nuclear envelope (Fig 2C), and this signal is reduced in the *nup42ΔFG nup159ΔFG* mutant reported to have decreased mRNP remodeling capacity (Fig 3B). Third, although GFP-dbp5^R369G^ associates with Mex67 as robustly as GFP-Dbp5 (Fig 2A), it does not accumulate in the nucleus of the *mex67-5* mutant (Fig 1D). This disconnect suggests that nuclear accumulation of Dbp5 is not reflective of its association with Mex67-bound mRNPs. Finally, Dbp5 does not accumulate in the nucleus when other mRNA export mutants are shifted to the non-permissive temperature, even though mRNA export is halted at a terminal step in these strains (Fig 1A). Therefore, the essential role of Dbp5 in remodeling mRNPs at the cytoplasmic face of the NPC does not require prior association in the nucleus.

Our data and our resulting model are consistent with a recent report that defined the nuclear export signal (NES) for Dbp5 and concluded that nuclear shuttling is not essential for mRNA export [33]. The discovery of an NES in Dbp5 establishes that nuclear export of Dbp5 itself is not dependent on mRNA export, in agreement with the lack of nuclear Dbp5 enrichment observed in *rat7-1* and *gle1-4* mutants (Fig 1). Indeed, we have observed rapid accumulation of Dbp5 in a strain that expresses the leptomycin B-sensitive *xpo1* allele. This strain has substantially slower kinetics of nuclear mRNA retention relative to NES-dependent export, supporting XpoI-dependent export of Dbp5. Another report has suggested that the majority of nuclear Dbp5 is not bound to mRNPs based on relatively rapid diffusion dynamics in the nucleus [69], consistent with a model supported by our data that Dbp5 does not stably interact with mRNPs prior to export. This is further supported by single-molecule imaging in *Chironomus tentans* that shows the majority of Dbp5 molecules approaching the NPC from the cytoplasm, and not from the nuclear side via export on mRNPs [70]. This agrees with the Dbp5-nup159ΔNTD fusion, which is anchored to the cytoplasmic face of the NPC and is viable. The viability of *DBP5-nup159ΔNTD* mutants is strikingly similar to the recent observation that fusion of Mex67 to the amino terminus of Nup116 is viable [69]. We did not test the viability of any other Dbp5 fusions, including fusion to NPC components on the nuclear face of the NPC, but we anticipate that localization to the cytoplasmic face is essential for remodeling at the terminal stage of mRNP export. Importantly, although the Dbp5-nup159ΔNTD fusion is sufficient for viability, the resulting strain displays a growth defect and minor mRNA export defect (S4 Fig). The reduced fitness might be the result of lost Dbp5 dynamics at the NPC, decreased Dbp5 abundance at the NPC, and/or loss of nuclear or cytoplasmic functions of Dbp5. Further studies are required to explore these possibilities.

Using a variety of approaches, we have also demonstrated that Dbp5 is recruited in proximity to Mex67 and Nab2 for interaction with RNA. Mex67 and Nab2 specifically co-precipitate with Dbp5 (potentially in a larger complex), whereas other RBPs do not (Fig 2A). The association of the co-isolated complex members are mediated via protein-protein interactions since the RNA binding capacity of Dbp5 is not required. It should be noted that this experiment was not performed using conditions designed to retain the full RNP interactome [71]. Instead, it is likely that some RNA degradation occurred even in the absence of RNase in our experiment, and complete mRNPs were not isolated, as evidenced by the lack of co-precipitated Cbp80 with GFP-Dbp5. This suggests that only proximal protein interactors were co-purified and Dbp5, Nab2, and Mex67 associate in a complex. Likewise, Dbp5 demonstrates a split Venus interaction with Nab2, but not with other RBPs (Fig 2C). Proximal associations between Dbp5 and Mex67-Mtr2 is corroborated by prior cross-linking-mass spectrometry studies that captured an Mtr2-Gle1 complex [72]. Although the RNA binding mutant dbp5^R369G^ associates with Mex67 and Nab2, RNA is still required for the Dbp5-Mex67 and Dbp5-Nab2 association. We interpret this result to indicate that Mex67-Mtr2 and/or Nab2 adopt conformations permissive for association with Dbp5 when bound to RNA, and we predict that Mex67-Mtr2 and/or Nab2 interaction with RNA is required for binding to Dbp5. However, we have not been able to observe interactions between Dbp5, Mex67-Mtr2, Nab2, and Gle1 with recombinant proteins in the presence of RNA, suggesting that additional factors including unknown proteins or post-translational modifications are required for Dbp5 association with Nab2 and/or Mex67.

Using bimolecular fluorescence complementation, an association between Dbp5 and Nab2 is observable at the NE with no increased nuclear signal above background (Fig 2C). We have interpreted this result to indicate that Dbp5 is recruited in proximity to Nab2 and Mex67 for remodeling at the NPC during the terminal steps of mRNA export. However, the BiFC signal only reports on close proximity of two proteins *in vivo*, not necessarily indicating a direct interaction. Additionally, the observed location of the Dbp5-VC Nab2-VN BiFC signal does not necessarily indicate the initial location of the Dbp5-Nab2 association. Due to the delay in fluorescence caused by chromophore maturation kinetics, it remains possible that the Dbp5-Nab2 association occurs first in the nucleus, and the Dbp5-VC Nab2-VN complex, stabilized by the complementation of the Venus fold, retains a steady-state localization at the NPC mediated by Dbp5 interaction with Nup159. Regardless, the viability of *DBP5-nup159ΔNTD* mutant suggests that nuclear association between Dbp5 and Nab2 or Mex67 is not essential and supports a model wherein these associations occur at the NPC. It is furthermore likely that the Dbp5-VC Nab2-VN fluorescent complementation signal overrepresents the native association due to the tendency of re-constituted fluorophores to remain associated, elongating the Nab2-Dbp5 association kinetics (S3C Fig). Indeed, we anticipate that the Nab2-Dbp5 and/or Mex67-Dbp5 association *in vivo* is transient and leads directly to mRNP remodeling. According to our model, once Nab2 and Mex67 are released from mRNA, they can no longer associate with Dbp5. It is also possible that the observed Dbp5-VC Nab2-VN association might occur during re-import of Nab2 and not necessarily in the context of an exporting mRNP. However, we did not observe a nuclear rim BiFC signal using the negative controls Cbp80 and Pab1, both of which are also shutting mRNA binding proteins. Further, specificity of Dbp5 association with Nab2, and not Pab1 or Cbp80, is further supported by our co-immunoprecipitation studies (Fig 2A).

Using this split-Venus signal as a reporter, we also observed an incomplete signal for Dbp5-Nab2 association around the NE under conditions where ribosome export is enhanced (Fig 3C), Based on quantification, the observed Nab2-Dbp5 BiFC association is reduced at NPCs adjacent to the nucleolus. We propose that this pattern of localization reflects specific cargo export through select NPCs under these conditions, perhaps related to the gene gating phenomenon [73,74]. Subsequent experiments can determine whether this selective export occurs in *nup60*, *mlp1*, or *pml39* mutants [60,61]. Alternatively, the proposed selective export could occur passively as ribosomes out-compete mRNA for export through nucleolar-associated NPCs.

The question remains as to why Dbp5 accumulates in the nucleus of *mex67-5* mutants and apparently interacts with RNA in the nucleus in an ATP-dependent manner (Fig 1D). It is possible that Dbp5 functions in resolving nuclear DNA:RNA hybrids, termed R-loops. The human DDX19B accumulates in the nucleus during DNA damage to resolve these transient transcription-dependent R-loops [32]. R-loops also form when RNA biogenesis/export factors of the THO and TREX complex are disrupted [75,76], so it is possible that in *S*. *cerevisiae* the *mex67-5* mutant results in R-loop formation that recruits Dbp5 to the nucleus. Through analysis of synthetic genetic interactions, the Cole lab concluded that Dbp5 has a function during mRNA transcription [77,78], but this role is not defined. The minor split Venus signal detected in the nucleus between Dbp5-VC and Cbp80-VN (Fig 2B) may be reflective of such a Dbp5 nuclear function. Nuclear accumulation of Dbp5 in *mex67-5* mutants might also reflect a function in tRNA maturation, as Mex67 has previously been linked to tRNA export [79]. Indeed, a role for Nab2 in tRNA surveillance has also been uncovered [80,81], and these Dbp5 and Nab2 tRNA functions might be related. Given the potential for a separable nuclear function by Dbp5/DDX19B and its established cytoplasmic role in translation termination [28,29,31], it seems clear that different subcellular pools of Dbp5 modulate mRNA metabolism at multiple stages in its lifecycle in independent manners. The loss of such auxiliary cytoplasmic/nuclear functions are likely responsible for the growth defects of *DBP5-nup159ΔNTD* compared to wild type strains (S4B Fig), although the viability of *dbp5Δ* strains expressing Dbp5-nup159ΔNTD demonstrates that such cytoplasmic/nuclear functions are not as critical as Dbp5’s role in mRNA export (Fig 4E). It remains to be determined if Dbp5 remodels Mex67 from the ribosomal stalk region of the 60S ribosomal subunit [82], where a role during ribosome export has been proposed [83].

Our data assert that Dbp5’s diverse functions are modulated by interaction partners that specify its actions in space and time. For example, if Dbp5 were recruited to interact with and kink mRNA in proximity to Mex67 and/or Nab2 prior to export, it would likely induce removal of these proteins before the transcript could cross the NPC. Instead, Mex67 and/or Nab2 bind Dbp5 while they are bound to RNA at the cytoplasmic face of the NPC, and Dbp5 interaction with mRNA here induces their release. Thus, Dbp5 mRNP remodeling effectively serves as a NPC transport “stop” signal. This transient Dbp5 interaction with RNA contrasts with eIF4AIII, the DEAD-box protein found at the core of the EJC [reviewed in 84]. Following splicing, eIF4AIII is recruited to bind RNA just upstream of the splice junction, and it scaffolds other EJC members that inhibit its ATPase activity so that it remains stably bound to RNA. No such inhibitor of Dbp5 ATPase activity has been uncovered to promote long-lived interaction with RNA. Instead, Dbp5 likely dynamically binds RNA, guided by distinct protein-protein interactions. Such diverse functions (RNA biogenesis, export, translation) have not been demonstrated for other DEAD-box proteins, making Dbp5 an ideal candidate for future studies directed at understanding how this class of proteins are regulated.

## Materials and methods

### Yeast strains and growth

S1 Table lists the yeast strains used in this study. Yeast genetic methods including mating, sporulation, dissection, and transformations were conducted according to standard procedures. Yeast strains were grown at indicated temperatures in either YPD (2% peptone, 2% dextrose, 1% yeast extract) or selective minimal media lacking appropriate amino acids and supplemented with 2% dextrose and 5-fluoroorotic acid (5-FOA; United States Biological) as needed at 1.0 mg/mL. For split Venus tagging, haploid strains were transformed with either VN:HIS3MX or VC:KANMX6 integration cassettes. For haploid strains where no integrations were confirmed, diploids were transformed, integration was confirmed by PCR and western blot, and strains were sporulated and dissected. No viable tagged haploids were recovered from these diploids.

### Vector construction

S2 Table lists the vectors used in this study. Vector cloning was performed either according to standard molecular biology strategies [85] or using Gibson Assembly (NEB). Restriction digest and DNA sequencing confirmed constructs.

### Live cell microscopy and *in situ* hybridization

Yeast strains were grown to mid-log phase (OD_600_ ~0.5) in–Leu or YPD media at the indicated temperatures, and where indicated, shifted to 37°C for 30min, shifted to 42°C for 10min, or thiolutin (Enzo) was added for indicated time points. Concentration of thiolutin was based on [59]. For Fig 3E, cells were grown to saturation overnight, diluted to early log phase (OD_600_ ~0.5) and grown for an additional 7hrs, and imaged. For live cell imaging, cultures were collected, re-suspended in synthetic complete media at the proper temperature, and imaged. For *in situ* hybridization, yeast were processed as described, hybridized with 1ng/μL Cy3-conjugated oligo d(T) or Alexa Flour 488-conjugated oligo d(T), stained with 0.1mg/mL DAPI to visualize the nucleus as described in [86]. Wide-field images were acquired using a microscope (BX50; Olympus) equipped with a motorized stage (Model 999000, Ludl), Olympus 100× NA1.3 UPlanF1 objective, and digital charge coupled device camera (Orca-R2; Hamamatsu). Images were processed with ImageJ (NIH) or Adobe Photoshop CS6.

Images for Fig 3C were quantified in Image J. Brightness and contrast were adjusted identically for all images. Using the line tool at 5 pixel thickness, a freehand line ROI was drawn around the nuclear envelope that either was adjacent or not adjacent to the Nop1-mCherry signal in a merged image. The mean grey value for the split Venus channel was calculated for the selected ROI as depicted in S3E Fig. Background fluorescence was measured by drawing an ROI next to the cell of interest. Background was subtracted, and the fluorescence of the region adjacent to the Nop1-mCherry signal was divided by the fluorescence of the region away from the Nop1-mCherry signal. One hundred cells from two biological replicates (two different days) were quantified. Data were plotted using Prism Graph Pad.

### Immunoprecipitation and Immunoblotting

Immunoprecipitation was adapted from [11]. 50mLs of wt strains transformed with GFP-Dbp5 wt and mutant vectors were grown to mid-log phase (OD_600_~0.5) in synthetic media, harvested, and lysed by bead beating (3x 2min) in lysis buffer (25 mM HEPES (pH 7.4), 150 mM KCl, 2 mM MgCl_2_, 0.1% NP-40, 1 mM PMSF, 1x cOmplete EDTA-free protease inhibitor (Roche)). Lysate was either treated with 0.5ug/uL Rnase A or water for 10min on benchtop, and incubated with polyclonal rabbit anti-GFP antibodies (ThermoFisher) and Protein A sepharose (GE) for 30min. Beads were washed 3x with lysis buffer, and bound protein was eluted in SDS loading buffer (20% glycerol, 4%SDS, 50mM Tris-HCl pH 6.8, 100mM DTT, 0.01% bromophenol blue). Proteins were then resolved by SDS-PAGE and blotted using affinity-purified rabbit anti-Dbp5 (ASW42) [28], affinity-purified rabbit anti-Nab2 (ASW 44) [20], affinity-purified rabbit anti-Cbp80 [87], affinity-purified chicken anti-Nup159-NTD (ASW 55) [44], affinity purified rabbit anti-Mex67-Mtr2 (ASW 52), affinity purified mouse anti-Pab1 [88], or affinity purified rabbit anti-Yra1 [89] antibodies. The anti-Mex67-Mtr2 antibody was generated against purified His6-Mtr2-Mex67 [57] and affinity purified using the same antigen with the assistance of the Vanderbilt Antibody and Protein Resource.

Whole-cell lysate immunoblotting was performed as in [90]). Cells were grown to mid-log phase, and equal OD_600_ units of cell numbers were collected by centrifugation. Cells were re-suspended in SDS loading buffer, lysed by vortexing with glass beads, and boiled 5min. Lysates were then separated by SDS-PAGE and blotted using the above-described antibodies or anti-yPgk1 (Invitrogen). Alexa Flour 650-, 700-, or 800- conjugated secondary antibodies (1:5000, Molecular Probes) were visualized with the Li-Cor Odyssey scanner (Lincoln, NE).

### FRAP microscopy

Fluorescence recovery after photobleaching experiments were performed on wt strain expressing GFP fusion proteins. Cells were analyzed using an inverted confocal microscope (LSM510 META; Carl Zeiss) equipped with a 63×/1.40 NA Plan Apochromat oil lens (Carl Zeiss) at room temperature and photographed using a charge-coupled device camera (AxioCam; Carl Zeiss). AxioVision version 3.0 SP software (Carl Zeiss) was used to acquire images. GFP signal was detected using a 488-nm excitation signal at 5% laser power. One prebleach and 20 post-bleach images were collected with two iterations averaged for each image. The bleach event was 15 iterations at 50% laser power with the indicated ROI. Normalized fluorescence intensity was calculated as in [91]: F(t)_norm_ = ((F(t)_ROI_−F_bkgd_) / (F(t)_cell_- F_bkgd_)) x ((F(i)_ROI_−F_bkgd_) / (F(t)_cell_- F_bkgd_)) where F_ROI_ is the signal of the ROI, F_cell_ is the signal from an adjacent cell (to control for photobleaching), F_bkgd_ is background signal, t is the time point, and i is the initial pre-bleach signal [91]. ImageJ was used to quantify the mean grey value, and values were averaged from 10 cells for each strain. Data were plotted using Prism Graph Pad.

## Supporting information

S1 Fig mRNA localization in indicated mutants. (A and B) Mutants display a poly(A)+ export defect. Indicated mutant strains were grown to mid-log phase (OD_600_~0.5) in YPD, shifted to 37°C for 30min, processed for *in situ* hybridization with an Alexa Flour 488-labeled oligo d(T) probe, stained with DAPI, and imaged by wide-field fluorescence microscopy. Scale bar, 5μm. Images were adjusted identically. (C) WT and mutant GFP-Dbp5 constructs are expressed to similar levels. Mutant *mex67-5* strains with *GFP-dbp5* vectors were grown to mid-log phase (OD_600_~0.5) in minimal media and lysed in SDS loading buffer. Lysates were resolved by SDS-PAGE and immunoblotted using the indicated antibodies. (D) GFP-Dbp5 localization in wt strains. WT strains with the indicated vectors were grown to mid-log phase (OD_600_~0.5) in YPD, shifted to 37°C for 30min, and imaged by wide-field live-cell direct fluorescence microscopy. Scale bar, 5μm. This localization has been previously reported [35]. Images were adjusted identically. (E) The poly(A)+ RNA export defect is not altered in *mex67-5* mutants with *GFP-dbp5* mutant vectors. Mutant strains with the indicated vectors were grown to mid-log phase (OD_600_~0.5) in YPD, shifted to 37°C for 30min, processed for *in situ* hybridization with a Cy3-labeled oligo d(T) probe, stained with DAPI, and imaged by wide-field fluorescence microscopy. Scale bar, 5μm. Images were adjusted identically.
(TIF)

S2 Fig Split-Venus tagged proteins are expressed. (A) Background fluorescence of haploid split-Venus tagged strains. Indicated strains were grown to mid-log phase (OD_600_~0.5) in YPD and imaged by wide-field live-cell direct fluorescence microscopy. Scale bar, 5μm. Images were adjusted identically to Fig 2C. (B) Western blot of VC and VN tagged proteins. Lysates from indicated strains were resolved by SDS-PAGE and immunoblotted using the indicated antibodies.
(TIF)

S3 Fig Reversibility and quantification of split Venus signal. (A) Inversion of split Venus images from Fig 3A. (B) Inversion of split Venus images from Fig 3B. (C) The Dbp5-VC and Nab2-VN split Venus signal is mildly disrupted when RNA export is halted. Indicated strains were grown to mid-log phase (OD_600_~0.5) at 23°C, thiolutin was added to a final concentration of 5μg/mL for indicated time points, and cells were imaged by wide-field live-cell direct fluorescence microscopy. All Venus images were adjusted identically. Scale bar, 5μm. Note that the Dbp5-VC and Nab2-VN BiFC signal is weaker when cells are grown in minimal medium (D) Thiolutin disrupts mRNA export activity. An integrated *DBP5-GFP* strain was transformed with the *nab2ΔRGG-mCherry* mRNA export reporter. Cells were grown to mid-log phase (OD_600_~0.5) at 23°C, thiolutin was added to a final concentration of 5μg/mL for indicated time points, and cells were imaged by wide-field live-cell direct fluorescence microscopy. Scale bar, 5μm. (E) Using Nop1-mCherry signal as a guide for NE adjacent to the nucleolus, free-hand lines were drawn in Image J, and mean grey value was determined for background, nucleolus, and non-nucleolus regions. Only cells with crescent-shaped nucleoli were quantified.
(TIF)

S4 Fig Growth of and mRNA localization in *DBP5-nup159ΔNTD* mutants. (A) GFP-Dbp5-nup159ΔNTD is expressed as a full-length protein. Lysates from indicated GFP-tagged strains were resolved by SDS-PAGE and immunoblotted using the indicated antibodies. Asterisks indicate full-length proteins visible with high exposure. Note that post-lysis degradation is common for unstructured FG-domain proteins. Note that full-length protein was not visible with the Dbp5 antibody. (B) *DBP5-nup159ΔNTD* has a temperature-sensitive growth defect. The indicated deletion strains (*dbp5Δ*, *nup159Δ*, or *nup159Δ dbp5Δ*) carrying the indicated vectors were grown to mid-log phase (OD_600_~0.5) in YPD at 23°C, fivefold serially diluted, and plated on YPD plates at the indicated temperature. (C) GFP tags do not affect growth of strains. The indicated strains were grown to mid-log phase (OD_600_~0.5) in YPD at 23°C, fivefold serially diluted, and plated on YPD plates at the indicated temperature. (D) Poly(A)+ localization in mutants. Indicated mutant strains were grown to mid-log phase (OD_600_~0.5) in YPD, shifted to 37°C for 30min, processed for *in situ* hybridization with a Cy3-labeled oligo d(T) probe, stained with DAPI, and imaged by wide-field fluorescence microscopy. Scale bar, 5μm. Images were adjusted identically. (E) Quantification of mRNA export defect of samples found in (D). At least eighty cells were scored for the accumulation of poly(A)+ signal in the nucleus from the indicated conditions.
(TIF)

S1 Table Strain table. The genotype and source of *S*. *cerevisiae* strains utilized in this study are listed. *KAN*^*R*^ denotes kanamycin resistance cassette.
(DOCX)

S2 Table Vector table. Table provides the description and source of plasmids utilized in this study.
(DOCX)
