# Supplementary figures and images for: Dbp5 associates with RNA-bound Mex67 and Nab2 and its localization at the nuclear pore complex is sufficient for mRNP export and cell viability
Source: PLoS Genet. 2020 Oct 1;16(10):e1009033. doi: 10.1371/journal.pgen.1009033 (PMC7553267; doi:10.1371/journal.pgen.1009033)

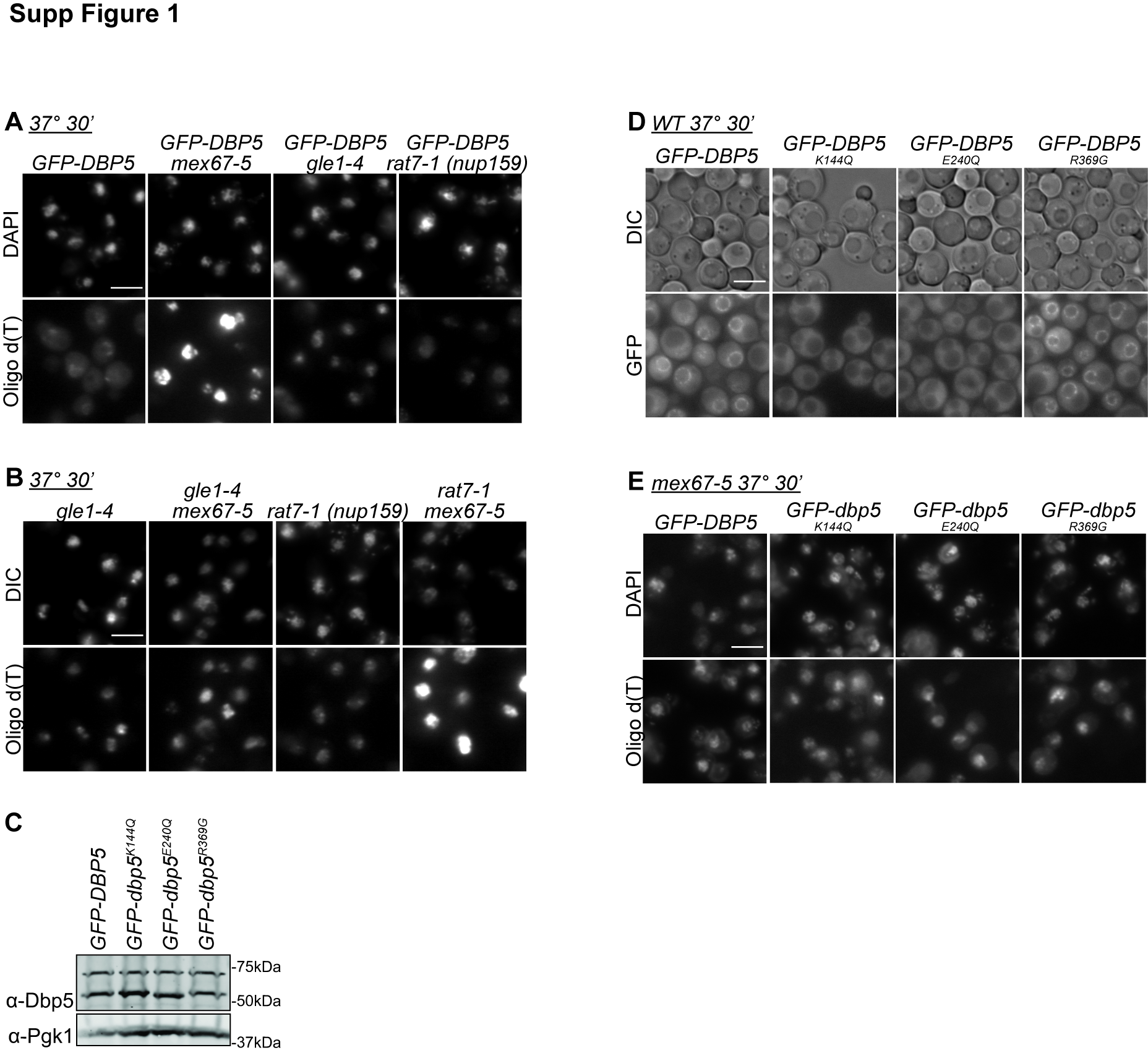

Supplement: S1 Fig — (A and B) Mutants display a poly(A)+ export defect. Indicated mutant strains were grown to mid-log phase (OD600~0.5) in YPD, shifted to 37°C for 30min, processed for in situ hybridization with an Alexa Flour 488-labeled oligo d(T) probe, stained with DAPI, and imaged by wide-field fluorescence microscopy. Scale bar, 5μm. Images were adjusted identically. (C) WT and mutant GFP-Dbp5 constructs are expressed to similar levels. Mutant mex67-5 strains with GFP-dbp5 vectors were grown to mid-log phase (OD600~0.5) in minimal media and lysed in SDS loading buffer. Lysates were resolved by SDS-PAGE and immunoblotted using the indicated antibodies. (D) GFP-Dbp5 localization in wt strains. WT strains with the indicated vectors were grown to mid-log phase (OD600~0.5) in YPD, shifted to 37°C for 30min, and imaged by wide-field live-cell direct fluorescence microscopy. Scale bar, 5μm. This localization has been previously reported [35]. Images were adjusted identically. (E) The poly(A)+ RNA export defect is not altered in mex67-5 mutants with GFP-dbp5 mutant vectors. Mutant strains with the indicated vectors were grown to mid-log phase (OD600~0.5) in YPD, shifted to 37°C for 30min, processed for in situ hybridization with a Cy3-labeled oligo d(T) probe, stained with DAPI, and imaged by wide-field fluorescence microscopy. Scale bar, 5μm. Images were adjusted identically. (TIF) [file pgen.1009033.s001.tif]

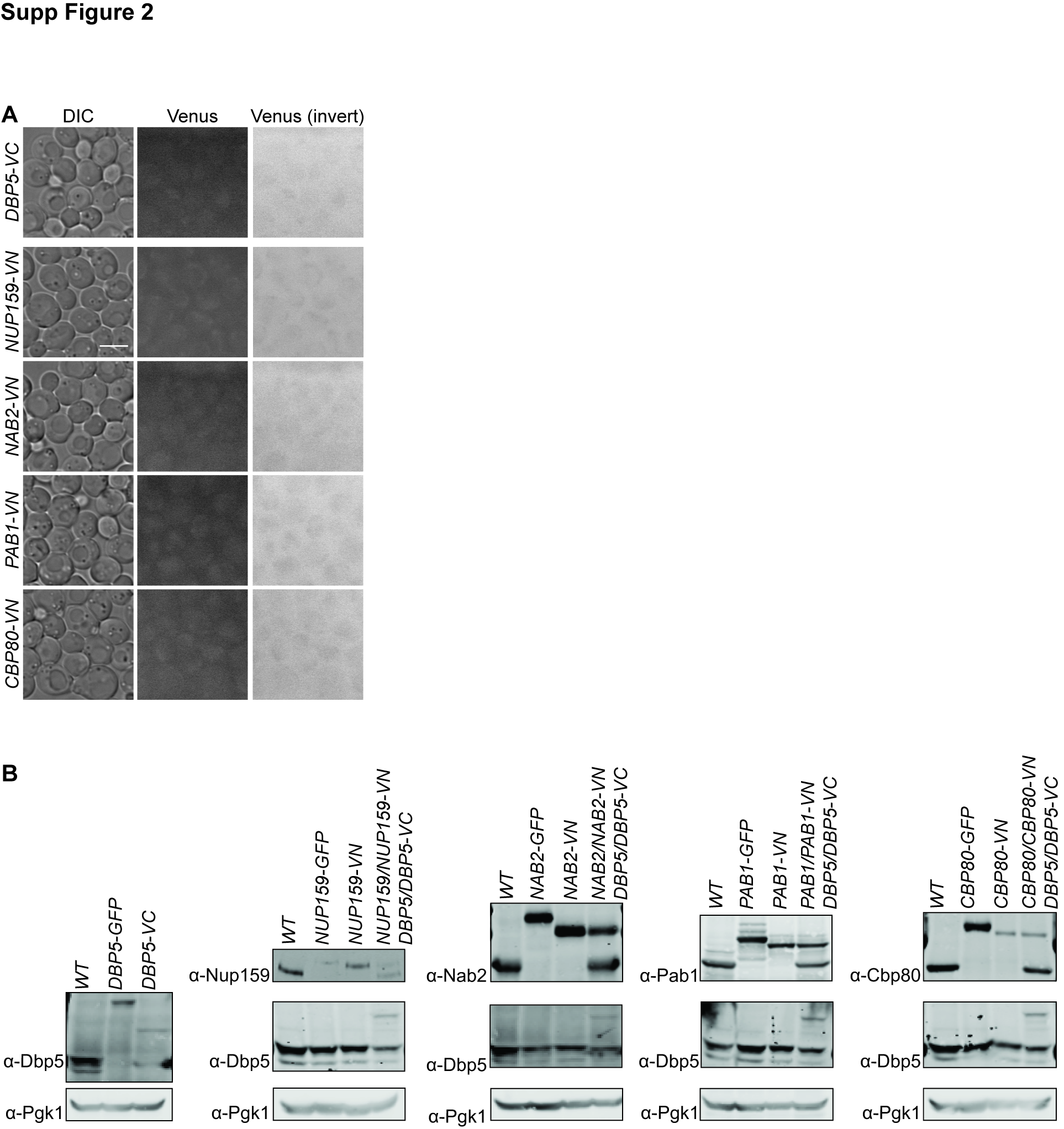

Supplement: S2 Fig — (A) Background fluorescence of haploid split-Venus tagged strains. Indicated strains were grown to mid-log phase (OD600~0.5) in YPD and imaged by wide-field live-cell direct fluorescence microscopy. Scale bar, 5μm. Images were adjusted identically to Fig 2C. (B) Western blot of VC and VN tagged proteins. Lysates from indicated strains were resolved by SDS-PAGE and immunoblotted using the indicated antibodies. (TIF) [file pgen.1009033.s002.tif]

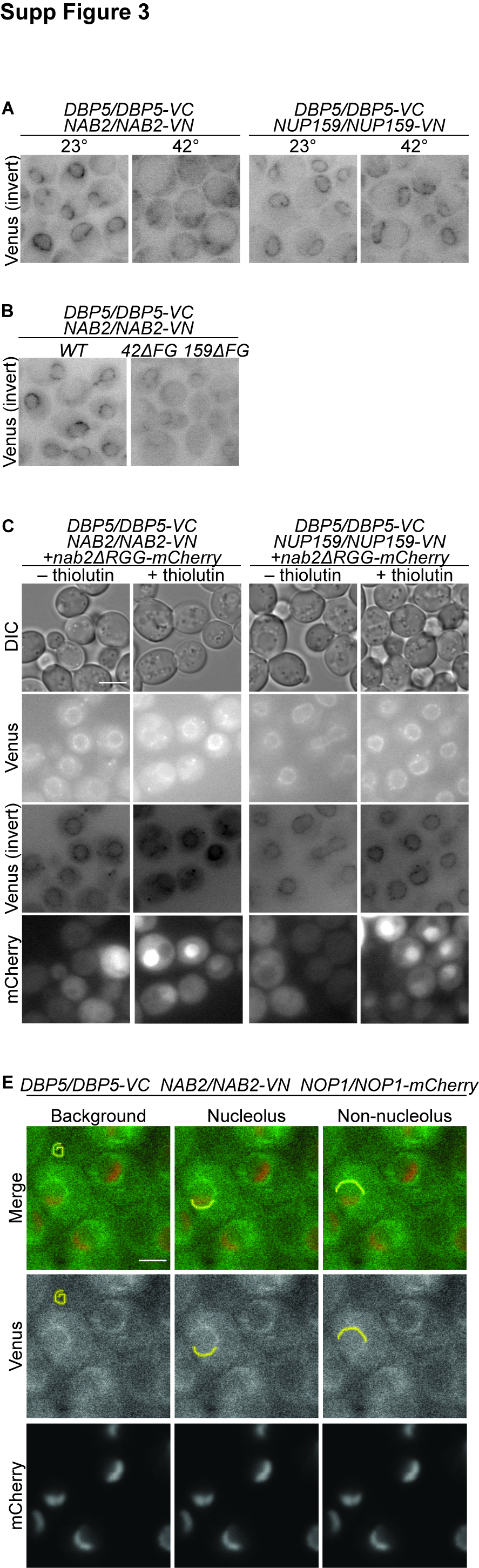

Supplement: S3 Fig — (A) Inversion of split Venus images from Fig 3A. (B) Inversion of split Venus images from Fig 3B. (C) The Dbp5-VC and Nab2-VN split Venus signal is mildly disrupted when RNA export is halted. Indicated strains were grown to mid-log phase (OD600~0.5) at 23°C, thiolutin was added to a final concentration of 5μg/mL for indicated time points, and cells were imaged by wide-field live-cell direct fluorescence microscopy. All Venus images were adjusted identically. Scale bar, 5μm. Note that the Dbp5-VC and Nab2-VN BiFC signal is weaker when cells are grown in minimal medium (D) Thiolutin disrupts mRNA export activity. An integrated DBP5-GFP strain was transformed with the nab2ΔRGG-mCherry mRNA export reporter. Cells were grown to mid-log phase (OD600~0.5) at 23°C, thiolutin was added to a final concentration of 5μg/mL for indicated time points, and cells were imaged by wide-field live-cell direct fluorescence microscopy. Scale bar, 5μm. (E) Using Nop1-mCherry signal as a guide for NE adjacent to the nucleolus, free-hand lines were drawn in Image J, and mean grey value was determined for background, nucleolus, and non-nucleolus regions. Only cells with crescent-shaped nucleoli were quantified. (TIF) [file pgen.1009033.s003.tif]

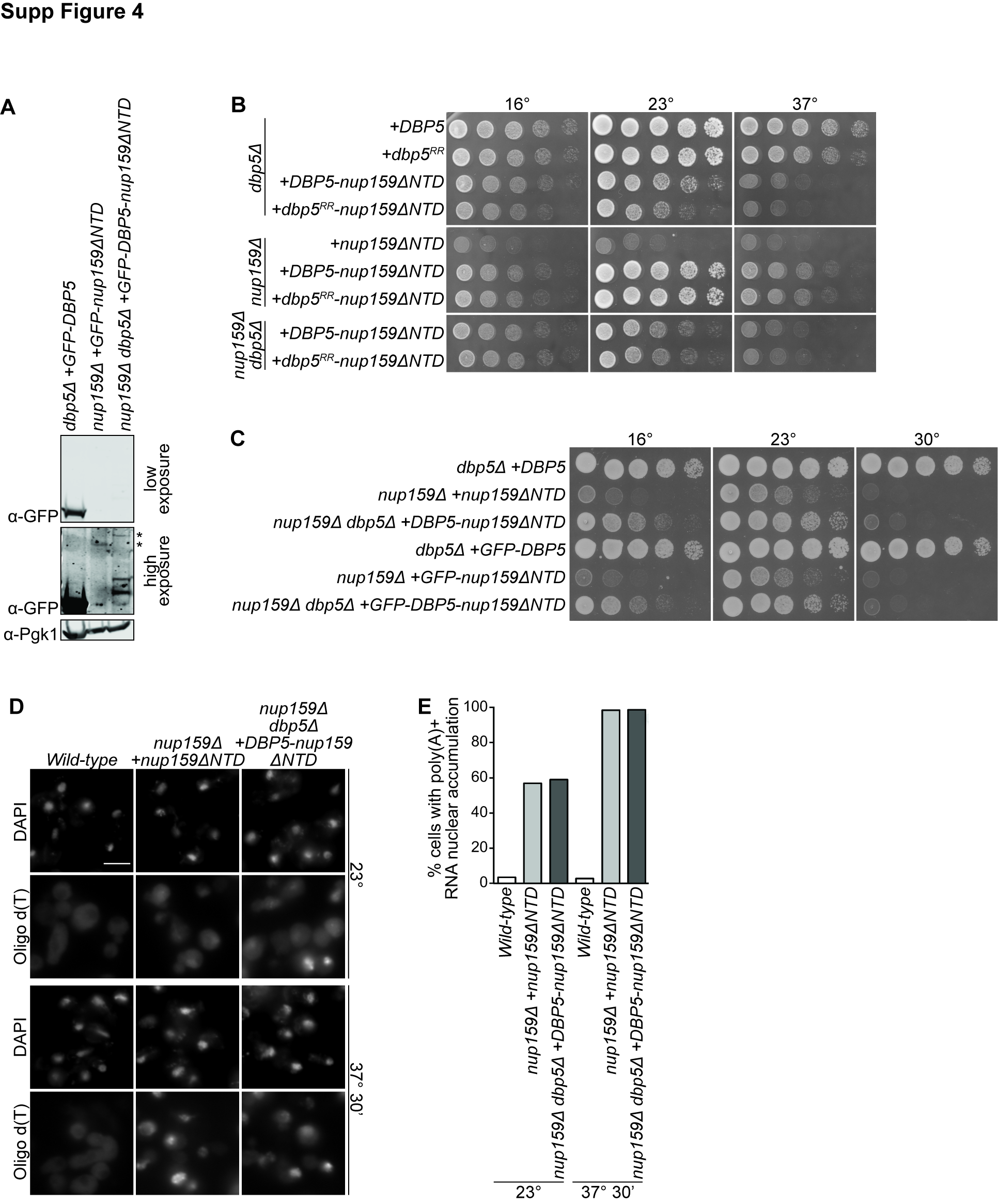

Supplement: S4 Fig — (A) GFP-Dbp5-nup159ΔNTD is expressed as a full-length protein. Lysates from indicated GFP-tagged strains were resolved by SDS-PAGE and immunoblotted using the indicated antibodies. Asterisks indicate full-length proteins visible with high exposure. Note that post-lysis degradation is common for unstructured FG-domain proteins. Note that full-length protein was not visible with the Dbp5 antibody. (B) DBP5-nup159ΔNTD has a temperature-sensitive growth defect. The indicated deletion strains (dbp5Δ, nup159Δ, or nup159Δ dbp5Δ) carrying the indicated vectors were grown to mid-log phase (OD600~0.5) in YPD at 23°C, fivefold serially diluted, and plated on YPD plates at the indicated temperature. (C) GFP tags do not affect growth of strains. The indicated strains were grown to mid-log phase (OD600~0.5) in YPD at 23°C, fivefold serially diluted, and plated on YPD plates at the indicated temperature. (D) Poly(A)+ localization in mutants. Indicated mutant strains were grown to mid-log phase (OD600~0.5) in YPD, shifted to 37°C for 30min, processed for in situ hybridization with a Cy3-labeled oligo d(T) probe, stained with DAPI, and imaged by wide-field fluorescence microscopy. Scale bar, 5μm. Images were adjusted identically. (E) Quantification of mRNA export defect of samples found in (D). At least eighty cells were scored for the accumulation of poly(A)+ signal in the nucleus from the indicated conditions. (TIF) [file pgen.1009033.s004.tif]
