# Supplementary material for: Dbp5 associates with RNA-bound Mex67 and Nab2 and its localization at the nuclear pore complex is sufficient for mRNP export and cell viability
Source: PLoS Genet. 2020 Oct 1;16(10):e1009033. doi: 10.1371/journal.pgen.1009033 (PMC7553267; doi:10.1371/journal.pgen.1009033)
Supplement: S1 Table — The genotype and source of S. cerevisiae strains utilized in this study are listed. KANR denotes kanamycin resistance cassette. (DOCX) [file pgen.1009033.s005.docx]

**S1 Table: Strain Table**

| Description | Genotype | Strain | Source |
| --- | --- | --- | --- |
| WT | *MATa ADE2 ADE3 TRP1 lys2 ura3-1 leu2-3,112 his3-11,15* | SWY2283 | (1) |
| *mex67-5* | *MATa MEX67::HIS3 pmex67-5/TRP1 trp1-1 ura3-1* | SWY2287 | This study |
| *DBP5-GFP* | *MATa DBP5-GFP:HIS3MX6*  *TRP1 lys2 ura3-1 leu2-3,112 his3-11,15* | SWY5249 | This study |
| *NUP159-GFP* | *MATa NUP159-GFP:HIS3MX6*  *TRP1 lys2 ura3-1 leu2-3,112 his3-11,15* | SWY5828 | This study |
| *NAB2-GFP* | *MATa NAB2-GFP:HIS3MX6*  *TRP1 lys2 ura3-1 leu2-3,112 his3-11,15* | SWY6290 | This study |
| *PAB1-GFP* | *MATa PAB1-GFP:HIS3MX6*  *TRP1 lys2 ura3-1 leu2-3,112 his3-11,15* | SWY6533 | This study |
| *CBP80-GFP* | *MATa CBP80-GFP:HIS3MX6*  *TRP1 lys2 ura3-1 leu2-3,112 his3-11,15* | SWY6531 | This study |
| *DBP5-GFP nup42ΔFG nup159ΔFG* | *MATa DBP5-GFP:HIS3MX6*  *HA-LoxP-nup42∆FG myc-LoxP-nup159∆FG*  *lys2 ura3-3 leu2-3,112 his3-11,15* | SWY5252 | This study |
| *NAB2-GFP nup42ΔFG nup159ΔFG* | *MATa NAB2-GFP:HIS3MX6*  *HA-LoxP-nup42∆FG myc-LoxP-nup159∆FG*  *lys2 ura3-3 leu2-3,112 his3-11,15* | SWY6291 | This study |
| *DBP5-VC* | *MATα DBP5-VC:KANMX6*  *ura3-1 his3-11,15 trp1-1 leu2-3,112* | SWY6465 | This study |
| *NUP159-VN* | *MATa NUP159-VN:HIS3MX6*  *lys2 ura3-1 leu2-3,112 his3-11,15* | SWY6463 | This study |
| *NAB2-VN* | *MATa NAB2-VN:HIS3MX6*  *lys2 ura3-1 leu2-3,112 his3-11,15* | SWY6461 | This study |
| *PAB1-VN* | *MATa PAB1-VN:HIS3MX6*  *lys2 ura3-1 leu2-3,112 his3-11,15* | SWY6462 | This study |
| *CBP80-VN* | *MATa CBP80-VN:HIS3MX6*  *lys2 ura3-1 leu2-3,112 his3-11,15* | SWY6464 | This study |
| *DBP5-VC NUP159-VN* | *MATα/a DBP5/DBP5-VC:KANMX6*  *NUP159-VN:HIS3MX6/NUP159*  *ura3-1/ura3-1 his3-11,15/his3-11,15 TRP1/trp1-1 leu2-3,112/leu2-3,112 lys2/LYS2* | SWY6474 | This study |
| *DBP5-VC NAB2-VN* | *MATα/a DBP5/DBP5-VC:KANMX6*  *NAB2-VN:HIS3MX6/NAB2*  *ura3-1/ura3-1 his3-11,15/his3-11,15 TRP1/trp1-1 leu2-3,112/leu2-3,112 lys2/LYS2* | SWY6472 | This study |
| *DBP5-VC PAB1-VN* | *MATα/a DBP5/DBP5-VC:KANMX6*  *PAB1-VN:HIS3MX6/PAB1 ura3-1/ura3-1*  *his3-11,15/his3-11,15 TRP1/trp1-1*  *leu2-3,112/leu2-3,112 lys2/LYS2* | SWY6473 | This study |
| *DBP5-VC CBP80-VN* | *MATα/a DBP5/DBP5-VC:KANMX6*  *CBP80-VN:HIS3MX6/CBP80 ura3-1/ura3-1*  *his3-11,15/his3-11,15 TRP1/trp1-1*  *leu2-3,112/leu2-3,112 lys2/LYS2* | SWY6475 | This study |
| *DBP5-VC NUP159-VN NOP1-mCherry* | *MATα/a DBP5/DBP5-VC:KANMX6*  *NUP159-VN:HIS3MX6/NUP159*  *NOP1/NOP1-mCherry:HygB ura3-1/ura3-1*  *his3-11,15/his3-11,15 TRP1/trp1-1*  *leu2-3,112/leu2-3,112 lys2/LYS2* | SWY6554 | This study |
| *DBP5-VC NAB2-VN NOP1-mCherry* | *MATα/a DBP5/DBP5-VC:KANMX6*  *NAB2-VN:HIS3MX6/NAB2*  *NOP1/NOP1-mCherry:HygB ura3-1/ura3-1*  *his3-11,15/his3-11,15 TRP1/trp1-1*  *leu2-3,112/leu2-3,112 lys2/LYS2* | SWY6551 | This study |
| *DBP5-VC NAB2-VN nup42ΔFG nup159ΔFG* | *MATα/a DBP5/DBP5-VC:KANMX6*  *NAB2-VN:HIS3MX6 HA-LoxP-nup42∆FG/HA-LoxP-nup42∆FG myc-LoxP-nup159∆FG/*  *myc-LoxP-nup159∆FG*  *ura3-1/ura3-1 his3-11,15/his3-11,15 TRP1/trp1-1*  *leu2-3,112/leu2-3,112 lys2/LYS2* | SWY6544 | This study |
| *nup159Δ shuffle strain* | *MATa nup159::KAN^R^ pNUP159/URA*  *ura3-1 his3-11,15 trp1-1 leu2-3,112* | SWY4303 | (2) |
| *dbp5Δ shuffle strain* | *MATa dbp5::KAN^R^ pDBP5/URA*  *ura3-1 his3-11,15 TRP leu2-3,112 lys2* | SWY5551 | This study |
| *dbp5Δ nup159Δ shuffle strain* | *MATa dbp5::KAN^R^ nup159::KAN^R^ pDBP5/URA pNUP159/URA ura3-1 his3-11,15 leu2-3,112* | SWY6145 | This study |
| *nup159Δ +nup159ΔN* | *MATa nup159::KAN^R^ pnup159ΔN/LEU*  *ura3-1 his3-11,15 trp1-1 leu2-3,112* | SWY6273 | This study |
| *nup159Δ +DBP5-nup159ΔN* | *MATa nup159::KAN^R^ pDBP5-nup159ΔN/LEU*  *ura3-1 his3-11,15 trp1-1 leu2-3,112* | SWY6274 | This study |
| *nup159Δ +dbp5^RR^-nup159ΔN* | *MATa nup159::KAN^R^ pdbp5^RR^-nup159ΔN/LEU*  *ura3-1 his3-11,15 trp1-1 leu2-3,112* | SWY6275 | This study |
| *dbp5Δ +DBP5* | *MATa dbp5::KAN^R^ pDBP5/LEU*  *ura3-1 his3-11,15 TRP leu2-3,112 lys2* | SWY6276 | This study |
| *dbp5Δ +dbp5^RR^* | *MATa dbp5::KAN^R^ pdbp5^RR^/LEU*  *ura3-1 his3-11,15 TRP leu2-3,112 lys2* | SWY6277 | This study |
| *dbp5Δ + DBP5-nup159ΔN* | *MATa dbp5::KAN^R^ pDBP5-nup159∆N/LEU*  *ura3-1 his3-11,15 TRP leu2-3,112 lys2* | SWY6278 | This study |
| *dbp5Δ +dbp5^RR^-nup159ΔN* | *MATa dbp5::KAN^R^ pdbp5^RR^-nup159∆N/LEU*  *ura3-1 his3-11,15 TRP leu2-3,112 lys2* | SWY6279 | This study |
| *dbp5Δ nup159Δ + DBP5-nup159ΔN* | *MATa dbp5::KAN^R^ nup159::KAN^R^*  *pDBP5-nup159∆N/LEU*  *ura3-1 his3-11,15 leu2-3,112* | SWY6280 | This study |
| *dbp5Δ nup159Δ + dbp5^RR^-nup159ΔN* | *MATa dbp5::KAN^R^ nup159::KAN^R^*  *pdbp5^RR^-nup159∆N/LEU*  *ura3-1 his3-11,15 leu2-3,112* | SWY6281 | This study |
| *nup159Δ +*  *GFP-nup159ΔN* | *MATa nup159::KAN^R^ pGFP-nup159∆N/LEU*  *ura3-1 his3-11,15 trp1-1 leu2-3,112* | SWY6282 | This study |
| *dbp5Δ + GFP-DBP5* | *MATa dbp5::KAN^R^ pGFP-DBP5/LEU*  *ura3-1 his3-11,15 leu2-3,112 lys2* | SWY6283 | This study |
| *nup159Δ dbp5Δ + GFP-DBP5-nup159ΔN* | *MATa dbp5::KAN^R^ nup159::KAN^R^*  *pGFP-DBP5-nup159∆N/LEU*  *ura3-1 his3-11,15 leu2-3,112* | SWY6285 | This study |
| *GFP-DBP5* | *MATa dbp5::KAN^R^ nup159::KAN^R^ pNUP159/TRP pGFP-DBP5/LEU ura3-1 his3-11,15 leu2-3,112* | SWY6563 | This study |
| *nup159ΔN*  *GFP-DBP5* | *MATa dbp5::KAN^R^ nup159::KAN^R^ pnup159∆N/LEU pGFP-DBP5/LEU ura3-1 his3-11,15 leu2-3,112* | SWY6564 | This study |
| *GFP-nup159ΔN* | *MATa dbp5::KAN^R^ nup159::KAN^R^*  *pGFP-nup159ΔN/LEU pDBP5/LEU ura3-1 his3-11,15 leu2-3,112* | SWY6565 | This study |
| *MEX67 DBP5-GFP* | *MATa mex67::HIS3 pMEX67/TRP*  *DBP5-GFP:HIS3MX6*  *ura3-1 his3-11,15 leu2-3,112 trp1-1* | SWY6069 | This study |
| *mex67-5 DBP5-GFP* | *MATa mex67::HIS3 pex67-5/TRP*  *DBP5-GFP:HIS3MX6*  *ura3-1 his3-11,15 leu2-3,112 trp1-1* | SWY6070 | This study |
| *gle1-4 DBP5-GFP* | *MATα gle1-4 DBP5-GFP:HIS3MX6*  *ura3-1 his3-11,15 leu2-3,112 trp1-1 ade2-1* | SWY6558 | This study |
| *rat7-1 DBP5-GFP* | *MATα rat7-1 (nup159) DBP5-GFP:HIS3MX6*  *ade2-1 ura3-1 his3-11,15 leu2-3,112 trp1-1* | SWY6557 | This study |
| *gle1-4* | *gle1-4 MEX67::HIS3 pMEX67/TRP1*  *ura3-1 his3-11,15 leu2-3,112 ade2-1* | SWY6597 | This study |
| *gle1-4 mex67-5* | *gle1-4 MEX67::HIS3 pmex67-5/TRP1*  *ura3-1 his3-11,15 leu2-3,112 ade2-1* | SWY6598 | This study |
| *rat7-1* | *rat7-1 MEX67::HIS3 pMEX67/TRP1*  *ura3-1 his3-11,15 leu2-3,112 ade2-1* | SWY6595 | This study |
| *rat7-1 mex67-5* | *rat7-1 MEX67::HIS3 pmex67-5/TRP1*  *ura3-1 his3-11,15 leu2-3,112 ade2-1* | SWY6596 | This study |

Supplemental References

1. Strawn LA, Shen T, Shulga N, Goldfarb DS, Wente SR. Minimal nuclear pore complexes define FG repeat domains essential for transport. Nat Cell Biol. 2004 Mar;6(3):197–206.

2. Adams RL, Terry LJ, Wente SR. Nucleoporin FG domains facilitate mRNP remodeling at the cytoplasmic face of the nuclear pore complex. Genetics. 2014 Aug;197(4):1213–24.
