# Supplementary material for: Dbp5 associates with RNA-bound Mex67 and Nab2 and its localization at the nuclear pore complex is sufficient for mRNP export and cell viability
Source: PLoS Genet. 2020 Oct 1;16(10):e1009033. doi: 10.1371/journal.pgen.1009033 (PMC7553267; doi:10.1371/journal.pgen.1009033)
Supplement: S2 Table — Table provides the description and source of plasmids utilized in this study. (DOCX) [file pgen.1009033.s006.docx]

**S2 Table: Vector Table**

| Vector | Description | Source |
| --- | --- | --- |
| pfa6a-VN-HIS3MX6 | For carboxy-terminal tagging with VN:HIS3MX3 | (1) |
| pfa6a-VC-KANMX6 | For carboxy-terminal tagging with VN:KANMX3 | (1) |
| pFA6a-GFP(s65t):HIS3MX6 | For carboxy-terminal tagging with GFP:HIS3MX3 | (2) |
| pBS35, mCherry:HygB | For carboxy-terminal tagging with mCherry:HygB | Yeast Resource Center |
| pRS314-TRP1-MEX67 | *MEX67/CEN/TRP1* | (3) |
| pRS314-TRP1-Mex67 | *mex67-5/CEN/TRP1* | (3) |
| pLG4 | *NUP159/CEN/URA3* | (4) |
| pCS834 | *DBP5/CEN/URA3* | (5) |
| pSW3353 | *DBP5/CEN/LEU2* | (6) |
| pSW3457 | *dbp5^RR^/CEN/LEU2* | (6) |
| pSW4231 | *nup159ΔNTD/CEN/LEU2* | This Study |
| pSW3647 | *NUP159/CEN/TRP1* | (7) |
| pSW4233 | *DBP5-nup159ΔNTD/CEN/LEU2* | This Study |
| pSW4234 | *dbp^RR^-nup159ΔNTD/CEN/LEU2* | This Study |
| pCS835 | *GFP-DBP5/CEN/LEU2* | (5) |
| pDBP5-K144E-GFP | *GFP-dbp5^K144E^/CEN/LEU2* | (8) |
| pDBP5-E240Q-GFP | *GFP-dbp5^E240Q^/CEN/LEU2* | (8) |
| pdbp5-R369G-GFP | *GFP-dbp5^R369G^/CEN/LEU2* | (8) |
| pSW4235 | *GFP-nup159ΔNTD/CEN/LEU2* | This Study |
| pSW4236 | *GFP-DBP5-nup159ΔNTD/CEN/LEU2* | This Study |
| pSW3842 | *nab2ΔRGG-mCherry/CEN/LEU2* | This Study |

Supplemental References

1. Webster BM, Colombi P, Jäger J, Lusk CP. Surveillance of nuclear pore complex assembly by ESCRT-III/Vps4. Cell. 2014 Oct 9;159(2):388–401.

2. Longtine MS, McKenzie A, Demarini DJ, Shah NG, Wach A, Brachat A, et al. Additional modules for versatile and economical PCR-based gene deletion and modification in Saccharomyces cerevisiae. Yeast Chichester Engl. 1998 Jul;14(10):953–61.

3. Segref A, Sharma K, Doye V, Hellwig A, Huber J, Lührmann R, et al. Mex67p, a novel factor for nuclear mRNA export, binds to both poly(A)+ RNA and nuclear pores. EMBO J. 1997 Jun 2;16(11):3256–71.

4. Gorsch LC, Dockendorff TC, Cole CN. A conditional allele of the novel repeat-containing yeast nucleoporin RAT7/NUP159 causes both rapid cessation of mRNA export and reversible clustering of nuclear pore complexes. J Cell Biol. 1995 May;129(4):939–55.

5. Snay-Hodge CA, Colot HV, Goldstein AL, Cole CN. Dbp5p/Rat8p is a yeast nuclear pore-associated DEAD-box protein essential for RNA export. EMBO J. 1998 May 1;17(9):2663–76.

6. Noble KN, Tran EJ, Alcázar-Román AR, Hodge CA, Cole CN, Wente SR. The Dbp5 cycle at the nuclear pore complex during mRNA export II: nucleotide cycling and mRNP remodeling by Dbp5 are controlled by Nup159 and Gle1. Genes Dev. 2011 May 15;25(10):1065–77.

7. Adams RL, Terry LJ, Wente SR. Nucleoporin FG domains facilitate mRNP remodeling at the cytoplasmic face of the nuclear pore complex. Genetics. 2014 Aug;197(4):1213–24.

8. Hodge CA, Tran EJ, Noble KN, Alcazar-Roman AR, Ben-Yishay R, Scarcelli JJ, et al. The Dbp5 cycle at the nuclear pore complex during mRNA export I: dbp5 mutants with defects in RNA binding and ATP hydrolysis define key steps for Nup159 and Gle1. Genes Dev. 2011 May 15;25(10):1052–64.
